# Down Syndrome Altered Cell Composition in Blood, Brain, and Buccal Swab Samples Profiled by DNA-Methylation-Based Cell-Type Deconvolution

**DOI:** 10.3390/cells12081168

**Published:** 2023-04-15

**Authors:** Ze Zhang, Hannah G. Stolrow, Brock C. Christensen, Lucas A. Salas

**Affiliations:** 1 Department of Epidemiology, Geisel School of Medicine at Dartmouth, Lebanon, NH 03756, USA; ze.zhang.gr@dartmouth.edu (Z.Z.); hannah.stolrow@dartmouth.edu (H.G.S.); brock.c.christensen@dartmouth.edu (B.C.C.); 2 Department of Molecular and Systems Biology, Geisel School of Medicine at Dartmouth, Lebanon, NH 03756, USA

**Keywords:** down syndrome, trisomy 21, DNA methylation, cell deconvolution, epigenetics, immune cell deconvolution, brain deconvolution

## Abstract

Down syndrome (DS) is a genetic disorder caused by an extra copy of chromosome 21 that presents developmental dysfunction and intellectual disability. To better understand the cellular changes associated with DS, we investigated the cell composition in blood, brain, and buccal swab samples from DS patients and controls using DNA methylation-based cell-type deconvolution. We used genome-scale DNA methylation data from Illumina HumanMethylation450k and HumanMethylationEPIC arrays to profile cell composition and trace fetal lineage cells in blood samples (DS N = 46; control N = 1469), brain samples from various regions (DS N = 71; control N = 101), and buccal swab samples (DS N = 10; control N = 10). In early development, the number of cells from the fetal lineage in the blood is drastically lower in DS patients (Δ = 17.5%), indicating an epigenetically dysregulated maturation process for DS patients. Across sample types, we observed significant alterations in relative cell-type proportions for DS subjects compared with the controls. Cell-type proportion alterations were present in samples from early development and adulthood. Our findings provide insight into DS cellular biology and suggest potential cellular interventional targets for DS.

## 1. Introduction

Down syndrome (DS) is a genetic disorder caused by the presence of an extra copy of chromosome 21 (trisomy 21). As the most common chromosomal abnormality in humans, DS affects approximately 1 in 700 live births in the US and 1 in 1000 live births worldwide [1,2]. DS is associated with a variety of physical and cognitive symptoms, including developmental delay, intellectual disability, distinct facial features, and an increased risk of specific medical conditions such as congenital heart disease [3]. DNA-methylation-based epigenetic clocks can accurately estimate biological age [4]. Epigenetic age acceleration occurs when an individual’s biological age is older than their chronological age [5]. Such a phenomenon is associated with an increased risk of various diseases [4,5]. Epigenetic age acceleration is a hallmark of developmental dysregulation in individuals with DS [6]. The increased risk of age-related diseases in the DS population, such as dementia and Alzheimer’s disease, is studied in order to be associated with epigenetic age acceleration [6,7]. Immune system abnormalities were also associated with DS. DS patients have an increased incidence of autoimmune disorders, such as celiac disease, thyroid disease, and type 1 diabetes, as well as an increased susceptibility to infectious diseases [8,9,10,11,12]. At the cellular level, individuals with DS have been shown to have reduced numbers of T and B lymphocytes and granulocytes in peripheral blood [13,14,15]. This functional immunosuppression may contribute to the increased susceptibility relative to infections and autoimmune disorders observed in DS. Furthermore, brain cell dysregulation in DS patients can lead to intellectual disability and other cognitive and behavioral symptoms [16]. Individuals with DS often experience a negative impact on their quality of life and overall health. One of the main difficulties in the DS population is oral health maintenance [17]. DS populations have a higher risk of oral diseases such as dental caries, periodontal disease, and abnormalities of the tongue and palate [17,18,19]. This increased risk is associated with various factors, including altered immune function, structural abnormalities of the teeth and jaws, and difficulty with oral hygiene self-care [17,18].

DNA methylation occurs when a methyl group is added to a cytosine base in a DNA molecule, typically at CpG sites. It is an epigenetic modification that plays a critical role in regulating gene expression and maintaining cellular identity [20]. Genome-wide DNA methylation arrays can be used to measure DNA methylation across hundreds of thousands of CpG sites. In DS, DNA methylation changes were largely found on euploid (non-21) chromosomes [14,21]. By comparing DNA methylation patterns in a mixture to those in a reference library of known cell types, it is possible to infer the relative proportions of each cell type in the mixture [22]. This method, known as DNA methylation deconvolution (or methylation cytometry), provides a standardized and cost-effective approach for assessing cell-type proportions and can easily be applied to archived samples [22]. Our group developed high-resolution DNA methylation deconvolution in the blood for profiling twelve immune cell types [23] and the brain for profiling seven major brain cell types [24]. Buccal swab deconvolution methods were also developed in previous studies by other research groups [25,26]. Furthermore, we devised a DNA methylation biomarker to trace cells of fetal origins in various tissues [27,28].

Understanding cell-type distributions in biospecimens from DS subjects can advance the understanding of DS pathophysiology and perhaps allow the consideration of cellular targets. Previous research investigated changes in deconvolved blood cell types in DS using DNA methylation but with limited cell types [14]. In this study, we comprehensively deconvolved blood, brain, and buccal swab samples during the various stages of life and compared cellular alterations in DS patients to normal populations using DNA methylation deconvolution.

## 2. Materials and Methods

### 2.1. Data Sets

This study utilized publicly available data sets from the Gene Expression Omnibus (GEO) and ArrayExpress consisting of DNA methylation microarray data from 1707 samples. The samples included blood, brain, and buccal swab samples from individuals with Down syndrome (DS) and their corresponding normal controls (Table 1). The blood samples consisted of 46 DS patients (age range: 0.5–43 years) and 1469 normal controls (age range: 0–94 years). The brain samples included 71 adult DS patients and 101 normal controls from various regions, including the cerebellum, frontal cortex, cerebrum, cerebellar cortex whole gray matter, frontal cortex whole gray matter, frontal cortex neuron, and frontal cortex glia. Buccal swab samples consisted of ten adult DS patients and ten normal controls.

### 2.2. DNA Methylation Data Processing

*GEOquery* (version 2.64.2, Sean Davis, Bethesda, Rockville, MD, USA) from *Bioconductor* in R was used to download DNA methylation microarray data and phenotype data from GEO [29]. The data from ArrayExpress was directly downloaded from the website. Methylation microarray IDAT files for data sets GSE174555 [30], GSE85042 [31], GSE87571 [32], GSE121633 [33], and E-MTAB-7069 [34] were processed using the *minfi* package (version 1.42.0, Martin Aryee, Boston, MA, USA) [35]. DNA methylation beta values were generated using the normal-exponential out-of-band (Noob) method for those data sets. The Noob preprocessing of the data was recommended for methylation-based deconvolution [23]. Average methylation beta values were directly used for data sets GSE36064 [36], GSE50586 [37], GSE52588 [38], GSE63347 [6], and GSE74486 [39], as the IDAT files were not available for those data sets.

### 2.3. Cell-Type Deconvolution

DNA methylation-based cell-type deconvolution methods were applied to estimate the cell composition across the samples. *FlowSorted.BloodExtended.EPIC* (version 0.99.0, Lucas Salas, Lebanon, NH, USA) was used to infer 12 immune cell proportions in whole blood samples, including basophils (Bas), eosinophils (Eos), neutrophils (Neu), monocytes (Mono), B naïve cells (Bnv), B memory cells (Bmem), CD4T naïve cells (CD4nv), CD4T memory cells (CD4mem), T regulatory cells (Treg), CD8T naïve cells (CD8nv), CD8T memory cells (CD8mem), and natural killer cells (NK) [23]. *HiBED* (version 0.99.0, Ze Zhang, Lebanon, NH, USA) was used to infer seven brain cell proportions in brain samples, including astrocytes, microglia, oligodendrocytes, GABAergic neurons (GABA), glutamatergic neurons (GLU), endothelial cells, and stromal cells [24]. *EpiDISH* (version 2.12.0, Shijie Zheng, Shanghai, China) was used to infer three buccal swab cell types, including immune cell, epithelial cell, and fibroblast [40]. Cells that arise from a fetal lineage in the early stages of life contain DNA methylation features that provide a memory trace of their fetal cell origin (FCO) [28]. The FCO DNA methylation signature was employed in all samples to estimate the proportion of cells that are of fetal lineages [27].

### 2.4. Data Analysis

Age is known to alter immune cell composition in a non-linear pattern. To account for age as an effect modifier between DS disease status and immune cell composition, the study population was stratified into three age groups (0–5, 10–18, and >18) for whole blood samples. We skipped groups 5–10, as no DS patients were identified in this age range. A series of multivariable linear regression models was applied in each age group to study the cell composition change between DS individuals and normal controls, adjusting for age and sex. Thirteen models were generated in total for whole blood samples in each group. The Loess smoothing method was used to depict the lifetime trajectories of FCO between DS individuals and normal controls. To account for the significant variations in cell composition and function across different regions in the human brain, brain samples were stratified by region and cell type. Seven strata were created in total, including the cerebellum, frontal cortex, cerebrum, cerebellar cortex whole gray matter, frontal cortex whole gray matter, frontal cortex neurons, and frontal cortex glia. A series of multivariable linear regression models was applied in each stratum to study the cell composition change between DS individuals and normal controls, adjusting for age and sex. Eight models were generated in total for whole blood samples in each stratum. Four multivariable linear regression models were developed for buccal swab samples to compare the cell composition between DS patients and normal controls, adjusting for age and sex. The false discovery rate (FDR) was calculated to account for multiple comparisons. An FDR of 0.05 was used as the statistical significance cut-off threshold. All analyses were performed using R version 4.2.0.

**Table 1:** Baseline characteristics of the data sets.

Sample	N	Mean Age (sd)	n Male (%)	Data Source
Blood				
Down Syndrome	46	17.2 (13.3)	29 (63)	GSE174555, GSE52588
Normal	1469	44.7 (23.7)	487 (33.2)	GSE174555, GSE52588, GSE121633, GSE174555, GSE36064, GSE52588, GSE85042, GSE87571, E-MTAB-7069
Brain ^#^				
Down Syndrome	71	48.3 (19)	45 (63.4)	GSE74486 *, GSE63347
Normal	101	49.8 (16)	41 (40.6)	GSE74486 *, GSE63347
Buccal Swab				
Down Syndrome	10	34.5 (6.8)	5 (50)	GSE50586
Normal	10	34.1 (6.1)	5 (50)	GSE50586
Total	1707			

^#^ Brain regions included the cerebellum, frontal cortex, cerebrum, cerebellar cortex whole gray matter, frontal cortex whole gray matter, frontal cortex neurons, and frontal cortex glia. * Horvath methylation age [41] was inferred using the *ENmix* package (version 1.32.0, Zongli Xu, Research Triangle Park, NC, USA) [42] due to the lack of age information.

## 3. Results

**Figure 1:**
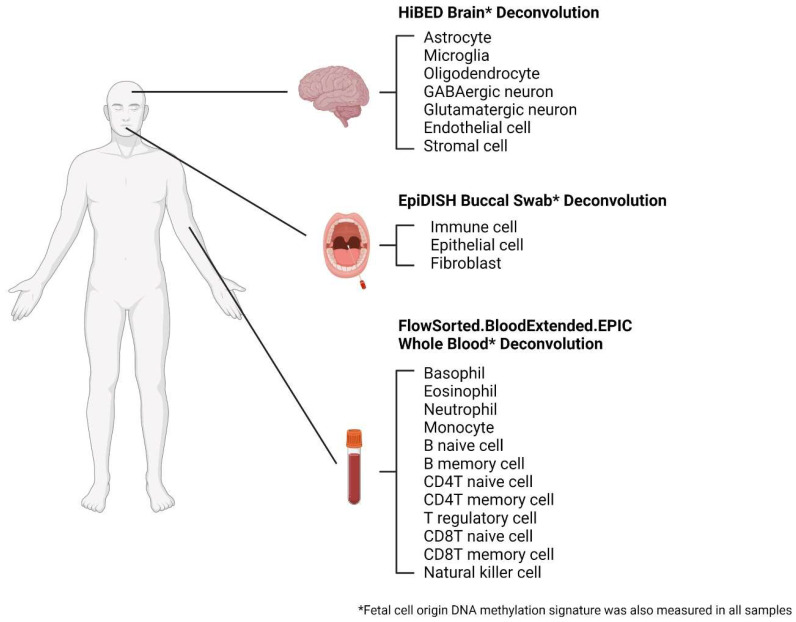
DNA-methylation-based cell-type deconvolution methods used to estimate cell proportions in this study (created with Biorender.com, last accessed on 13 April 2023).

DNA methylation was used to deconvolve cell types in blood, brain, and buccal swab samples from individuals with DS and normal controls, as shown in Figure 1. The summary statistics of the deconvolved cell types are shown in Supplementary Tables S1–S3 for blood, brain, and buccal swab samples, respectively.

### 3.1. DS-Altered Fetal-to-Adult Cell Lineage Transition in Blood

**Figure 2:**
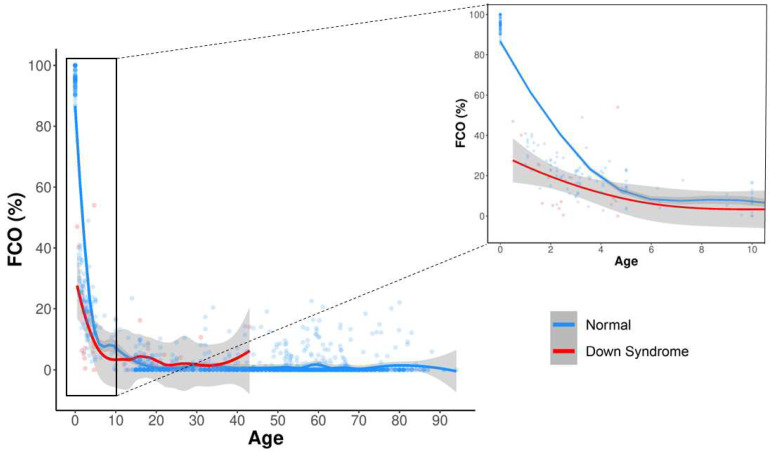
The FCO trajectories in DS and normal populations described an accelerated process of fetal-to-adult lineage cell transition, especially in DS individuals under 10 years old.

**Figure 3:**
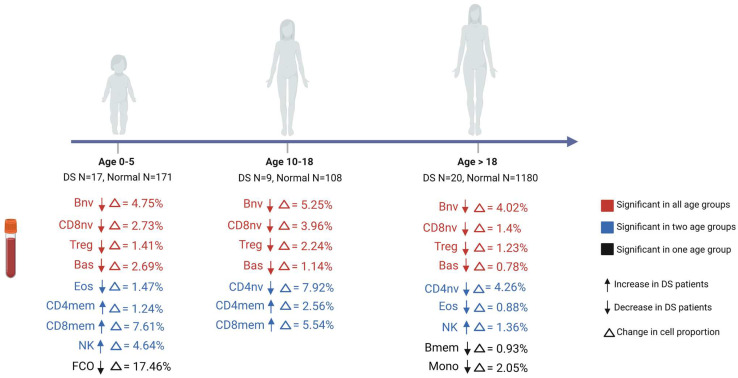
Whole blood immune cell composition changes in proportions in DS patients compared to normal controls in different life stages (created with Biorender.com, accessed on 13 April 2023).

Fetal lineage cells are reminiscent of their origins in DNA methylation [28]. The FCO DNA methylation signature was devised to trace cells that are of a fetal lineage [27]. It estimates the proportion of cells in a mixture of cell types that are of fetal origin [27,28]. In our study, the FCO trajectories in DS and control populations described an accelerated process of fetal-to-adult lineage cell transition, with a more pronounced effect in DS individuals under ten years old (Figure 2), indicating a significantly earlier completion of the fetal-to-adult lineage cell transition in DS patients. In the 0–5 age group, we observed a significantly lower proportion of cells harboring the FCO signature (Δ = 17.46%; FDR = $5.7\times 10^{-4}$) (Figure 3). The results suggest an epigenetically dysregulated maturation process for DS patients in the early stages of life.

### 3.2. DS-Altered Blood Cell Composition

Whole blood immune cell composition differences in DS patients compared to normal controls are shown in Figure 3. We observed decreases in Bas (Δ = 2.69%, FDR = 0.026), Bnv (Δ = 4.75%, FDR = $5.7\times 10^{-4}$), CD8nv (Δ = 2.73%, FDR = 0.011), Eos (Δ = 1.47%, FDR = 0.034), and Treg (Δ = 1.41%, FDR = 0.04) and increases in CD4mem (Δ = 1.24%, FDR = $3\times 10^{-5}$), CD8mem (Δ = 7.61%, FDR = $1.2\times 10^{-12}$), and NK cells (Δ = 4.64%, FDR = $2.4\times 10^{-6}$) in DS patients compared to normal controls. In the 10–18 age group, we observed significant decreases in Bas (Δ = 1.14%, FDR = 0.024), Bnv (Δ = 5.25%, FDR = $1.6\times 10^{-7}$), CD4nv (Δ = 7.92%, FDR = 1.4E-10), CD8nv (Δ = 3.96%, FDR = $1.7\times 10^{-4}$), and Treg (Δ = 2.24%, FDR = 0.008) and increases in CD4mem (Δ = 2.56%, FDR = 0.0011) and CD8mem (Δ = 5.54%, FDR = $6.5\times 10^{-4}$) in DS patients compared to normal controls. In the >18 age group, we observed significant decreases in Bas (Δ = 0.78%, FDR = $3\times 10^{-4}$), Bmem (Δ = 0.93%, FDR = 0.022), Bnv (Δ = 4.02%, FDR = $3\times 10^{-14}$), CD4nv (Δ = 4.26%, FDR = $5.9\times 10^{-6}$), CD8nv (Δ = 1.4%, FDR = $8.7\times 10^{-6}$), Eos (Δ = 0.88%, FDR = 0.043), Mono (Δ = 2.05%, FDR = $7\times 10^{-5}$), and Treg (Δ = 1.23%, FDR = $7\times 10^{-5}$) and increases in NK cells (Δ = 1.36%, FDR = 0.026) in DS patients compared to normal controls. The complete model statistics summary of the multivariable linear regression models is shown in Supplementary Tables S4–S6. The direct comparison and distribution of blood immune cell proportions between DS and normal control populations by age group are shown in Supplementary Figures S1–S3. Across the three age groups, Bnv, CD8nv, Bas, and Treg demonstrated consistent decreases in DS populations compared to normal controls. The increases in CD4mem, CD8mem, and NK cells and the decreases in Eos and CD4nv were observed in two out of three age groups.

### 3.3. DS-Altered Brain Cell Composition

**Figure 4:**
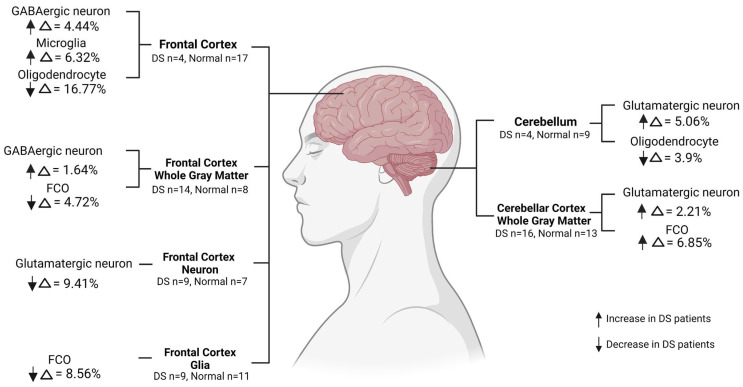
Brain cell composition changes in proportions in DS patients compared to normal controls in different brain regions (created with Biorender.com, accessed on 13 April 2023).

Brain cell composition changes in DS patients compared to normal controls are demonstrated in Figure 4. In the frontal cortex region, we observed significant increases in GABA (Δ = 4.4%, FDR = 0.003) and microglia (Δ = 6.32%, FDR = 0.029) and a significant decrease in oligodendrocytes (Δ = 16.77%, FDR = 0.029) in DS patients compared to normal controls. In the frontal cortex whole gray matter, we observed a significant increase in GABA (Δ = 1.64%, FDR = 0.011) and a significant decrease in FCO cells (Δ = 4.72%, FDR = 0.032) in DS patients compared to normal controls. A significant decrease in GLU was observed in frontal cortex neurons (Δ = 9.41%, FDR = 0.036) in DS patients. A significant increase in FCO cells (Δ = 8.56%, FDR = 0.038) in DS patients in frontal cortex glia was observed. In the cerebellum region, a significant increase in GLU (Δ = 5.06%, FDR = 0.0047) and a significant decrease in oligodendrocytes (Δ = 3.9%, FDR = 0.03) in DS patients compared to normal controls. In the cerebellar cortex’s whole gray matter, we observed significant increases in GLU (Δ = 2.21%, FDR = 0.014) and cells bearing the FCO signature (Δ = 6.85%, FDR = 0.009) in DS patients compared to normal controls. No significant difference in cell composition was found in the fetal cerebrum between DS patients and normal controls. The complete model’s statistical summary of multivariable linear regression models is shown in Supplementary Tables S7–S13. The direct comparison of brain cell proportions between DS and normal control populations by brain region is shown in Supplementary Figures S4–S10. In summary, the DS-altered brain cell composition varies vastly by brain region. Consistently, the frontal cortex region showed higher GABA levels, whereas GLU levels were higher in the cerebellar regions.

### 3.4. DS-Altered Buccal Swab Cell Composition

**Figure 5:**
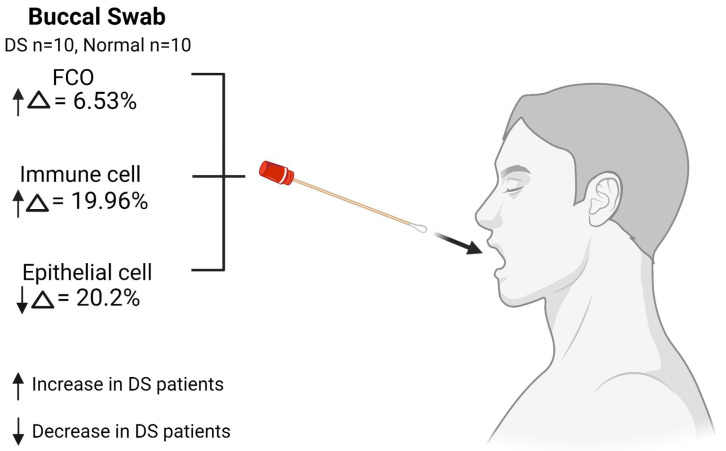
Buccal swab cell composition changes in proportions in DS patients compared to normal controls (created with Biorender.com, accessed on 13 April 2023).

In buccal swab samples, we observed significant increases in FCO cells (Δ = 6.53%, FDR = 0.013) and immune cells (Δ = 19.96%, FDR = 0.013) and a significant decrease in epithelial cells (Δ = 20.2%, FDR = 0.013) in DS patients compared to normal controls (Figure 5). The complete model statistics summary is shown in Supplementary Table S14. The direct comparison of the buccal swab cell proportions between DS and normal control populations is shown in Supplementary Figure S11.

## 4. Discussion

Individuals with Down syndrome (DS) exhibit developmental impairments, which are characterized by delays in both physical and intellectual growth [3]. The DNA methylation-based epigenetic aging clock is an effective tool for tracking the biological maturation process, as reflected by the difference between the epigenetic and chronological ages [4]. Previous studies reported significant age acceleration in DS patients compared to normal populations in whole blood, brain, and buccal swab samples from adults and the blood of newborns, indicating a malfunctioning aging process in DS patients [6,7]. The fetal cell origin (FCO) DNA methylation signature is a methylation-based deconvolution method used to trace the cells that are of a fetal lineage [27]. Although it tracks with age, FCO specifically captures the fetal-to-adult cell lineage transition across tissues in early-stage development [27]. Our study described an accelerated fetal-to-adult blood cell lineage transition in DS patients in the early stages of life. The average discrepancy in the FCO cells between DS patients and normal children ages 0–5 is 17.5%. The drastic loss of blood FCO cells in early childhood aligns with previously observed epigenetic age acceleration in the DS population. The FCO difference between DS patients and normal controls in blood did not persist in the population over the age of 10, as the fetal-to-adult cell lineage transition is near completion with a low FCO level (around 0) by that time. Unlike blood, the adult human brain retains a fetal cell compartment (around 10% of the whole brain) [28]. The FCO cell difference between DS and normal populations in the brain varied by location in our study. While the reduction in FCO cells was observed in frontal cortical regions in DS patients, the FCO cell level was higher in cerebellar regions. Although the function of the fetal cell compartment in the brain is yet to be studied, the change in direction in the FCO cell difference between DS patients and normal controls in frontal cortical regions and cerebellar regions implies potential distinct biological pathways to DS physical and intellectual characteristics impacted by the FCO cells in different brain regions. The frontal lobe and cerebellum are two distinct brain regions with different functional roles. The frontal cortex is responsible for high-level cognitive and executive functions, such as the production and comprehension of language [43], whereas the cerebellum is responsible for coordinating and fine-tuning movements, e.g., posture and balance [44]. We hypothesize that in the frontal cortical regions, the fetal-to-adult cell lineage transition was overcommitted, contributing to cognitive and executive dysfunctions in DS patients, whereas in the cerebellar regions, the fetal-to-adult cell lineage transition was incomplete, interfering with movement and coordination in DS patients. Further studies are needed to establish the functional link between regional cells and the FCO compartment in the brain. Distinguished from the FCO change in blood and brain, FCO cells retained a significantly higher level in buccal swab samples from adult DS patients than in normal controls. Profound changes in epigenetic profiles characterize buccal swab samples from DS patients, resulting in accelerated aging, differential DNA methylation patterns, and immune cell alterations [6,13,37]. We posit that the FCO cells retained in buccal swabs in adult DS patients may have biological implications in those epigenetic-related changes. However, future studies are needed to establish such relationships.

DS individuals have an increased risk of immune-related disorders, including autoimmune disorders [8], immune deficiencies [12], and hematological malignancies [45]. Previous studies reported cell composition altered by DS in neonatal blood, including lower proportions of B lymphocytes, CD4T lymphocytes, and granulocytes and a higher proportion of NK cells [14]. However, prior results are limited by the number of cell types that can be measured in blood. In this study, we were able to achieve a higher resolution of immune cell profiling in DS blood using DNA-methylation-based cell-type deconvolution [23]. Our extensive profiling of the peripheral immune cells provides insights into DS-related immune malfunctions. Abnormal eosinophil and basophil differentiation in DS and their relationship with DS-related leukemia were reported in previous studies [46,47,48]. We identified significant reductions in basophil and eosinophil proportions in DS patients. This result expanded the prior finding of a reduced granulocyte proportion in DS individuals. The DS population is known for increased susceptibility to infection, especially recurrent respiratory infections with increased severity and a prolonged course of the disease [48,49]. Expanding on the previous findings of reduced B and T lymphocytes in DS patients, we characterized the decreases in B-naïve, CD4T-naïve, and CD8T-naïve cell proportions and the increases in CD4T memory and CD8T memory cell proportions in DS patients. The observation of a general decrease in naïve cell proportions and an increase in memory cell proportions sheds light on the immune defect in DS patients specifically related to infection susceptibility. Tregs are critical immune cells for maintaining immune tolerance and preventing autoimmune diseases [50]. Studies have shown that reduced Treg numbers can contribute to the development of autoimmune diseases [50,51]. We observed a significant decrease in Treg proportions in DS patients in this study, which is consistent with the knowledge that people with DS have an increased risk of developing autoimmune disorders such as celiac disease, type 1 diabetes, and rheumatoid arthritis. The comprehensive profiling of DS-altered peripheral immune cells by our study could potentially lead to identifying interventional targets for DS patients to achieve normal immunity.

Morphological and cellular changes in DS brains were reported in previous studies [13,52,53]. Multiple cell-type irregularities in the brain were found to be associated with DS pathology, including those in neurons, astrocytes, oligodendrocytes, microglia, and endothelial cells [13,53]. Our study reported multiple brain cell proportion changes by brain region in DS patients. GABA neurons and GLU neurons are two distinct types of neurons that deploy different neurotransmitters to communicate with other neurons in the brain. GABA neurons release inhibitory neurotransmitters that repress the activity of other neurons, while GLU neurons release excitatory neurotransmitters that increase the activity of other neurons [54,55]. Our study reported increased GABA neuron levels in frontal cortical regions and decreased GLU neuron levels in cerebellar cortical regions in DS patients. The increased GABA neuron level in frontal cortical regions indicates suppressive neural activity in the area that is responsible for cognitive processes in DS patients. The increased GLU neuron level in cerebellar regions indicates excitatory neural activity in the area that is responsible for motor coordination and balance in DS patients. DS brains were characterized by hypomyelination and a reduced number of oligodendrocytes [53,56]. Consistent with the previous findings, we observed a decrease in DS patients’ oligodendrocytes in the frontal cortex and cerebellum. Microglia appeared to be more proliferative and active in DS populations in previous studies [53,57]. We revealed a significant increase in microglial cell proportions in the frontal cortex in DS patients. The illustration of the regional brain cell alteration by DS provides insight into the potential pathways of cellular modification on cognitive and physical malfunctions in DS patients.

Individuals with DS are at an increased risk of oral diseases and struggle with oral health maintenance [17]. A substantial shift in buccal swab cells in DS patients was observed in our study, with a significant increase in immune cell proportion and a significant decrease in epithelial cell proportion. We hypothesize that such a shift is largely attributed to the difficulties of oral hygiene maintenance for DS patients. However, future studies are needed to investigate the relationship between oral health behavior and buccal swab cell composition in DS patients. Buccal swab samples are non-invasive and commonly used in DS patients for genetic testing. Buccal swab deconvolved cell types can potentially serve as a DS oral health tracking biomarker.

While our study comprehensively profiled the DS-altered cell composition in blood, brain, and buccal swab samples, we recognize some limitations. First, although DNA methylation-based cell-type deconvolution yields accurate cell proportion estimation, a whole blood count is needed to track the numbers of the cells. The number of cells is usually tracked with proportional changes in cells, but there could be discrepancies between cell count change and cell proportion change. Future work with measurements of whole cell count in tissues in combination with cell-type deconvolution is ideal for studying cell count changes in the DS population. Second, only blood samples in this study span different stages of life. Brain samples are predominantly adult, with only five fetal cerebrum samples. Future studies on depicting FCO changes in brain tissues are of major interest for tracking the fetal brain cell maturation process in DS patients. Third, brain samples are limited to the frontal cortical and cerebellar regions. Future studies to profile DS-altered cell composition in other brain regions, such as the hippocampus and basal ganglia, are necessary to more comprehensively study brain cellular pathways to DS behavioral change. Fourth, the copy number variation was not corroborated in the quality control process, as some data sets only provided preprocessed methylation beta matrices. Fifth, although previous studies highlighted DS-associated DNA methylation changes in euploid (non-21) chromosomes, whether aneuploids can cause methylation measurement variations was not studied. However, the deconvolution methods rely lightly on the chr 21 CpGs (<1%). We believe our findings are solid in this study. Finally, we were not able to identify blood samples from DS patients ages 5–10. Future data to fill this gap are necessary to make sure the results are replicable within this age group.

## 5. Conclusions

We comprehensively profiled the DS-altered cell composition in blood, brain, and buccal swab samples using DNA-methylation-based cell-type deconvolution. Our findings offer valuable insight into the cellular pathobiology of DS and can potentially serve as a basis for developing new therapeutic strategies to improve the health and wellbeing of individuals with DS. Identifying specific cell types in the blood, brain, and buccal swabs that are altered in individuals with DS provides opportunities for developing targeted interventions to restore normal immunity, cognitive and motor behavior, and oral health.

## Data Availability

All data sets used in this study are publicly available on Gene Expression Omnibus (GEO) and ArrayExpress. The accession numbers are GSE174555, GSE52588, GSE121633, GSE174555, GSE36064, GSE52588, GSE85042, GSE87571, GSE74486, GSE63347, GSE50586, and E-MTAB-7069.

## Supplementary Materials

The following supporting information can be downloaded at: https://www.mdpi.com/article/10.3390/cells12081168/s1, Supplementary Tables S1–S14 presented summaries of multiple variable linear regression model outputs. Supplementary Figures S1–S11 presented boxplots of cell proportions between DS patients and normal controls. All Supplementary Materials are summarized in a single PDF file.

## Abbreviations

DS: Down syndrome; Bas: basophil; Eos: eosinophil; Neu: neutrophil; Mono: monocyte; Bnv: B naïve cell; Bmem: B memory cell; CD4nv: CD4T naïve cell; CD4mem: CD4T memory cell; Treg: T regulatory cell; CD8nv: CD8T naïve cell; CD8mem: CD8T memory; NK: natural killer cell; GABA: GABAergic neuron (GABA); GLU: glutamatergic neuron; GEO: Gene Expression Omnibus.

## References

[1] Mai C.T., Isenburg J.L., Canfield M.A., Meyer R.E., Correa A., Alverson C.J., Lupo P.J., Riehle-Colarusso T., Cho S.J., Aggarwal D. (2019). National population-based estimates for major birth defects, 2010–2014. Birth Defects Res..

[2] Rodrigues M., Nunes J., Figueiredo S., de Campos A.M., Geraldo A.F. (2019). Neuroimaging assessment in Down syndrome: A pictorial review. Insights Imaging.

[3] Antonarakis S.E., Skotko B.G., Rafii M.S., Strydom A., Pape S.E., Bianchi D.W., Sherman S.L., Reeves R.H. (2020). Down syndrome. Nat. Rev. Dis. Prim..

[4] Bell C.G., Lowe R., Adams P.D., Baccarelli A.A., Beck S., Bell J.T., Christensen B.C., Gladyshev V.N., Heijmans B.T., Horvath S. (2019). DNA methylation aging clocks: Challenges and recommendations. Genome Biol..

[5] Mendelson M.M. (2018). Epigenetic Age Acceleration: A Biological Doomsday Clock for Cardiovascular Disease?. Circ. Genom. Precis. Med..

[6] Horvath S., Garagnani P., Bacalini M.G., Pirazzini C., Salvioli S., Gentilini D., Di Blasio A.M., Giulina C., Tung S., Vinters H.V. (2015). Accelerated epigenetic aging in Down syndrome. Aging Cell.

[7] Xu K., Li S., Muskens I.S., Elliott N., Myint S.S., Pandey P., Hansen H.M., Morimoto L.M., Kang A.Y., Ma X. (2022). Accelerated epigenetic aging in newborns with Down syndrome. Aging Cell.

[8] Ferrari M., Stagi S. (2021). Autoimmunity and Genetic Syndromes: A Focus on Down Syndrome. Genes.

[9] Amr N.H. (2018). Thyroid disorders in subjects with Down syndrome: An update. Acta Biomed..

[10] Du Y., Shan L.-F., Cao Z.-Z., Feng J.-C., Cheng Y. (2017). Prevalence of celiac disease in patients with Down syndrome: A meta-analysis. Oncotarget.

[11] Anwar A.J., Walker J.D., Frier B.M. (1998). Type 1 diabetes mellitus and Down’s syndrome: Prevalence, management and diabetic complications. Diabet. Med..

[12] Ram G., Chinen J. (2011). Infections and immunodeficiency in Down syndrome. Clin. Exp. Immunol..

[13] Créau N. (2012). Molecular and Cellular Alterations in Down Syndrome: Toward the Identification of Targets for Therapeutics. Neural Plast..

[14] Muskens I.S., Li S., Jackson T., Elliot N., Hansen H.M., Myint S.S., Pandey P., Schraw J.M., Roy R., Anguiano J. (2021). The genome-wide impact of trisomy 21 on DNA methylation and its implications for hematopoiesis. Nat. Commun..

[15] Thilaganathan B., Tsakonas D., Nicolaides K. (1993). Abnormal fetal immunological development in Down’s syndrome. BJOG Int. J. Obstet. Gynaecol..

[16] Lott I.T. (2012). Neurological phenotypes for Down syndrome across the life span. Prog. Brain Res..

[17] Elrefadi R., Beaayou H., Herwis K., Musrati A. (2022). Oral health status in individuals with Down syndrome. Libyan J. Med..

[18] Gupta S., Goud E.S.S., Gulati S., Agrawal A., Pani P., Nishant K., Pattnaik S. (2021). Implications of Down’s syndrome on oral health status in patients: A prevalence-based study. J. Fam. Med. Prim. Care.

[19] Deps T.D., Angelo G.L., Martins C., Paiva S.M., Pordeus I., Borges-Oliveira A.C. (2015). Association between Dental Caries and Down Syndrome: A Systematic Review and Meta-Analysis. PLoS ONE.

[20] Bogdanović O., Lister R. (2017). DNA methylation and the preservation of cell identity. Curr. Opin. Genet. Dev..

[21] Sailani M.R., Santoni F.A., Letourneau A., Borel C., Makrythanasis P., Hibaoui Y., Popadin K., Bonilla X., Guipponi M., Gehrig C. (2015). DNA-Methylation Patterns in Trisomy 21 Using Cells from Monozygotic Twins. PLoS ONE.

[22] Titus A.J., Gallimore R.M., Salas L.A., Christensen B.C. (2017). Cell-type deconvolution from DNA methylation: A review of recent applications. Hum. Mol. Genet..

[23] Salas L.A., Zhang Z., Koestler D.C., Butler R.A., Hansen H.M., Molinaro A.M., Wiencke J.K., Kelsey K.T., Christensen B.C. (2022). Enhanced cell deconvolution of peripheral blood using DNA methylation for high-resolution immune profiling. Nat. Commun..

[24] Zhang Z., Wienke J.K., Kelsey K.T., Koestler D.C., Molinaro A.M., Pike S.C., Karra P., Christensen B.C., Salas L.A. (2023). Hierarchical deconvolution for extensive cell type resolution in the human brain using DNA methylation. Res. Sq..

[25] Zheng S.C., Webster A., Dong D., Feber A., Graham D.G., Sullivan R., Jevons S., Lovat L., Beck S., Widschwendter M. (2018). A novel cell-type deconvolution algorithm reveals substantial contamination by immune cells in saliva, buccal and cervix. Epigenomics.

[26] Theda C., Hwang S.H., Czajko A., Loke Y.J., Leong P., Craig J.M. (2018). Quantitation of the cellular content of saliva and buccal swab samples. Sci. Rep..

[27] Salas L.A., Wiencke J.K., Koestler D.C., Zhang Z., Christensen B.C., Kelsey K.T. (2018). Tracing human stem cell lineage during development using DNA methylation. Genome Res..

[28] Zhang Z., Wiencke J.K., Koestler D.C., Salas L.A., Christensen B.C., Kelsey K.T. (2019). Absence of an embryonic stem cell DNA methylation signature in human cancer. BMC Cancer.

[29] Davis S., Meltzer P.S. (2007). GEOquery: A bridge between the Gene Expression Omnibus (GEO) and BioConductor. Bioinformatics.

[30] Naumova O.Y., Lipschutz R., Rychkov S.Y., Zhukova O.V., Grigorenko E.L. (2021). DNA Methylation Alterations in Blood Cells of Toddlers with Down Syndrome. Genes.

[31] Maschietto M., Bastos L.C., Tahira A.C., Bastos E.P., Euclydes V.L.V., Brentani A., Fink G., de Baumont A., Felipe-Silva A., Francisco R.P.V. (2017). Sex differences in DNA methylation of the cord blood are related to sex-bias psychiatric diseases. Sci. Rep..

[32] Johansson A., Enroth S., Gyllensten U. (2013). Continuous Aging of the Human DNA Methylome Throughout the Human Lifespan. PLoS ONE.

[33] Kurushima Y., Tsai P.-C., Castillo-Fernandez J., Alves A.C., Moustafa J.S.E.-S., Le Roy C., Spector T.D., Ide M., Hughes F.J., Small K.S. (2019). Epigenetic findings in periodontitis in UK twins: A cross-sectional study. Clin. Epigenetics.

[34] Pérez R.F., Santamarina P., Tejedor J.R., Urdinguio R.G., Álvarez-Pitti J., Redon P., Fernández A.F., Fraga M.F., Lurbe E. (2019). Longitudinal genome-wide DNA methylation analysis uncovers persistent early-life DNA methylation changes. J. Transl. Med..

[35] Aryee M.J., Jaffe A.E., Corrada-Bravo H., Ladd-Acosta C., Feinberg A.P., Hansen K.D., Irizarry R.A. (2014). Minfi: A flexible and comprehensive Bioconductor package for the analysis of Infinium DNA methylation microarrays. Bioinformatics.

[36] Alisch R.S., Barwick B.G., Chopra P., Myrick L.K., Satten G.A., Conneely K.N., Warren S.T. (2012). Age-associated DNA methylation in pediatric populations. Genome Res..

[37] Jones M.J., Farré P., McEwen L.M., MacIsaac J.L., Watt K., Neumann S.M., Emberly E., Cynader M.S., Virji-Babul N., Kobor M.S. (2013). Distinct DNA methylation patterns of cognitive impairment and trisomy 21 in down syndrome. BMC Med. Genom..

[38] Bacalini M.G., Gentilini D., Boattini A., Giampieri E., Pirazzini C., Giuliani C., Fontanesi E., Scurti M., Remondini D., Capri M. (2015). Identification of a DNA methylation signature in blood cells from persons with Down Syndrome. Aging.

[39] Mendioroz M., Do C., Jiang X., Liu C., Darbary H.K., Lang C.F., Lin J., Thomas A., Abu-Amero S., Stanier P. (2015). Trans effects of chromosome aneuploidies on DNA methylation patterns in human Down syndrome and mouse models. Genome Biol..

[40] Zheng S.C., Breeze C.E., Beck S., Teschendorff A.E. (2018). Identification of differentially methylated cell types in epigenome-wide association studies. Nat. Methods.

[41] Horvath S. (2013). DNA methylation age of human tissues and cell types. Genome Biol..

[42] Xu Z., Niu L., Li L., Taylor J.A. (2016). ENmix: A novel background correction method for Illumina HumanMethylation450 BeadChip. Nucleic Acids Res..

[43] Goyal N., Siddiqui S.V., Chatterjee U., Kumar D., Siddiqui A. (2008). Neuropsychology of prefrontal cortex. Indian J. Psychiatry.

[44] Manto M., Bower J.M., Conforto A.B., Delgado-García J.M., Da Guarda S.N.F., Gerwig M., Habas C., Hagura N., Ivry R.B., Mariën P. (2012). Consensus paper: Roles of the cerebellum in motor control—The diversity of ideas on cerebellar involvement in movement. Cerebellum.

[45] Xavier A.C., Ge Y., Taub J.W. (2009). Down syndrome and malignancies: A unique clinical relationship: A paper from the 2008 william beaumont hospital symposium on molecular pathology. J. Mol. Diagn..

[46] Maroz A., Stachorski L., Emmrich S., Reinhardt K., Xu J., Shao Z., Käbler S., Dertmann T., Hitzler J., Roberts I. (2013). GATA1s induces hyperproliferation of eosinophil precursors in Down syndrome transient leukemia. Leukemia.

[47] Suda T., Suda J., Miura Y., Hayashi Y., Eguchi M., Tadokoro K., Saito M. (1985). Clonal analysis of basophil differentiation in bone marrow cultures from a Down’s syndrome patient with megakaryoblastic leukemia. Blood.

[48] Kusters M.A., Gemen E.F., Verstegen R.H.J., Wever P.C., de Vries E. (2010). Both normal memory counts and decreased naive cells favor intrinsic defect over early senescence of Down syndrome T lymphocytes. Pediatr. Res..

[49] Dieudonné Y., Uring-Lambert B., Jeljeli M.M., Gies V., Alembik Y., Korganow A.-S., Guffroy A. (2020). Immune Defect in Adults With Down Syndrome: Insights Into a Complex Issue. Front. Immunol..

[50] Kondělková K., Vokurková D., Krejsek J., Borská L., Fiala Z., Andrýs C. (2010). Regulatory T cells (Treg) and their roles in immune system with respect to immunopathological disorders. Acta Med..

[51] Dejaco C., Duftner C., Grubeck-Loebenstein B., Schirmer M. (2006). Imbalance of regulatory T cells in human autoimmune diseases. Immunology.

[52] Mrak R.E., Griffin W.S. (2004). Trisomy 21 and the brain. J. Neuropathol. Exp. Neurol..

[53] Becker L., Mito T., Takashima S., Onodera K. (1991). Growth and development of the brain in Down syndrome. Prog. Clin. Biol. Res..

[54] Huang Z.J., Paul A. (2019). The diversity of GABAergic neurons and neural communication elements. Nat. Rev. Neurosci..

[55] Zhou Y., Danbolt N.C. (2014). Glutamate as a neurotransmitter in the healthy brain. J. Neural Transm..

[56] Reiche L., Küry P., Göttle P. (2019). Aberrant Oligodendrogenesis in Down Syndrome: Shift in Gliogenesis?. Cells.

[57] García O., Flores-Aguilar L. (2022). Astroglial and microglial pathology in Down syndrome: Focus on Alzheimer’s disease. Front. Cell. Neurosci..

