# Supplementary material for: Down Syndrome Altered Cell Composition in Blood, Brain, and Buccal Swab Samples Profiled by DNA-Methylation-Based Cell-Type Deconvolution
Source: Cells. 2023 Apr 15;12(8):1168. doi: 10.3390/cells12081168 (PMC10136493; doi:10.3390/cells12081168)
Supplement: Supplementary file 1 [file cells-12-01168-s001.zip › cells-2338562-supplementary.pdf]

Supplementary Materials

Down Syndrome Altered Cell Composition in Blood, Brain, and Buccal Swab Samples Profiled by DNA Methylation-Based Cell Type Deconvolution

Table of Contents

Supplementary Table 1. Summary statistics of the deconvolved cell types in blood samples 3

Supplementary Table 2. Summary statistics of the deconvolved cell types in brain samples 4

Supplementary Table 3. Summary statistics of the deconvolved cell types in buccal swab samples 5

Supplementary Table 4. Multiple variable linear regression model outputs for blood cell change with DS adjusting for sex and age in the age group 0-5 6

Supplementary Table 5. Multiple variable linear regression model outputs for blood cell change with DS adjusting for sex and age in the age group 10-18 7

Supplementary Table 6. Multiple variable linear regression model outputs for blood cell change with DS adjusting for sex and age in the age group >18 8

Supplementary Table 7. Multiple variable linear regression model outputs for brain cell change with DS adjusting for sex and age in the frontal cortex 9

Supplementary Table 8. Multiple variable linear regression model outputs for brain cell change with DS adjusting for sex and age in the frontal cortex whole gray matter 10

Supplementary Table 9. Multiple variable linear regression model outputs for brain cell change with DS adjusting for sex and age in the frontal cortex neuron 11

Supplementary Table 10. Multiple variable linear regression model outputs for brain cell change with DS adjusting for sex and age in the frontal cortex glia 12

Supplementary Table 11. Multiple variable linear regression model outputs for brain cell change with DS adjusting for sex and age in the cerebellum 13

Supplementary Table 12. Multiple variable linear regression model outputs for brain cell change with DS adjusting for sex and age in the cerebellar cortex whole gray matter 14

Supplementary Table 13. Multiple variable linear regression model outputs for brain cell change with DS adjusting for sex and age in the fetal cerebrum 15

Supplementary Table 14. Multiple variable linear regression model outputs for buccal swab cell change with DS adjusting for sex and age........ 16

Supplementary Figure 1. Boxplots on blood cell differences between DS and normal groups in the age group 0-5 17

Supplementary Figure 2. Boxplots on blood cell differences between DS and normal groups in the age group 10-18 18

Supplementary Figure 3. Boxplots on blood cell differences between DS and normal groups in the age group >18 19

Supplementary Figure 4. Boxplots on brain cell differences between DS and normal groups in the frontal cortex 20

Supplementary Figure 5. Boxplots on brain cell differences between DS and normal groups in the frontal cortex whole gray matter 21

Supplementary Figure 6. Boxplots on brain cell differences between DS and normal groups in the frontal cortex neuron 22

Supplementary Figure 7. Boxplots on brain cell differences between DS and normal groups in the frontal cortex glia 23

Supplementary Figure 8. Boxplots on brain cell differences between DS and normal groups in the cerebellum 24

Supplementary Figure 9. Boxplots on brain cell differences between DS and normal groups in the cerebellar cortex whole gray matter 25

Supplementary Figure 10. Boxplots on brain cell differences between DS and normal groups in the fetal cerebrum 26

Supplementary Figure 11. Boxplots on brain cell differences between DS and normal groups in buccal swabs 27

	FCO (%)		Bas (%)		Bmem (%)		Bnv (%)		CD4mem (%)		CD4nv (%)		CD8mem (%)		CD8nv (%)		Eos (%)		Mono (%)		Neu (%)		NK (%)		Treg (%)	
	DS	Normal	DS	Normal	DS	Normal	DS	Normal	DS	Normal	DS	Normal	DS	Normal	DS	Normal	DS	Normal	DS	Normal	DS	Normal	DS	Normal	DS	Normal
Age 0-5																										
Minium	0	2.748715	0	0	0	0	1.11	2.04	0	0	2.02	3.25	0	0	0.15	0	0	0	3.01	2.09	14.16	5.75	3.89	0.18	0	0
1st Quantile	5.645124	20.72018	0	1.04	0.26	0	4.77	9.61	0	0	7.86	11.455	4.77	0	1.53	2.845	0	0	5.77	5.965	24.1	25.895	9.15	3.57	0	0
Median	8.73312	40.51849	0	3.05	0.65	0.65	6.11	12.06	0	0	12.03	14.48	10.05	0	3.74	5.84	0	0.68	6.57	7.26	37.52	37.4	10.05	5.39	0.88	0
Mean	16.56755	57.09609	0.08	3.950643	0.814118	0.852749	7.610588	12.59708	1.784118	0.256959	12.95882	15.31766	11.46882	2.471111	4.453529	6.187836	0.594118	1.69731	7.008824	7.53538	37.22	38.07924	10.38706	6.192515	1.685882	2.336082
3rd Quantile	19.21125	95.18282	0	4.365	1.46	1.275	9.45	15.26	3.5	0	17.11	18.625	21.52	4.385	7.58	9.03	0	2.48	8.61	9.07	45.11	49.23	11.56	8.36	2.87	5.11
Maximum	54.03032	100	0.81	32.42	1.73	5.05	19.25	25.83	7.91	4.4	29.98	36.38	22.87	18.29	11.34	21.93	6.04	14.76	10.24	16.99	81.32	74.55	17.92	20.87	5.82	11.82
Age 5-18																										
Minium	0	0	0	0	0.25	0	0	1.4	0.46	0	0	3.41	4.73	0	0	0	0	0	3.74	3.53	33.12	20.44	4.97	1.55	0	0
1st Quantile	0	0	0	0	0.61	0.205	0	5.015	2.32	0	1.12	7.7375	10.07	4.23	0	2.005	0	0	7.16	7.025	54.69	44.24	6.38	3.8675	0	0.7075
Median	1.645377	0	0	0.76	0.88	0.88	0	6.785	4.33	1.105	1.79	9.625	10.91	6.08	0	3.615	0	0	7.51	7.915	59.12	50.89	7.27	5.505	0	1.735
Mean	4.070368	2.623423	0	1.128519	0.823333	0.938148	1.707778	6.932222	4.455556	1.884444	1.837778	9.73537	12.28222	6.74	0.086667	4.026574	0.323333	1.0425	7.267778	8.187222	56.05444	50.37296	7.431111	5.815556	0	2.232315
3rd Quantile	6.85494	3.557201	0	1.675	0.97	1.4025	1.98	8.45	6.1	3.24	2.61	11.355	16.71	8.1075	0	5.5025	0	1.6375	8.52	9.305	60.65	55.58	8.54	7.06	0	2.845
Maximum	16.23255	23.45742	0	6.2	1.29	3.41	9.62	15.36	9.45	7.49	4.15	26.99	20.22	22.29	0.53	12.2	2.91	8.5	9.48	15.71	65.46	76.43	10.08	18.59	0	12.24
Age >18																										
Minium	0	0	0	0	0	0	0	0	0	0	0	0	3.74	0	0	0	0	0	0	1.37	46.77	9.89	2.62	0	0	0
1st Quantile	0	0	0	0	0.2525	1.13	0	1.41	2.71	3.84	0	1.4	7.6025	5.78	0	0	0	0	4.7475	6.53	53.9975	47.98	5.7875	3.33	0	0
Median	1.97E-16	0	0	0.05	0.485	1.88	0	2.79	4.91	6.44	1.695	4.89	9.795	9.04	0	0	0	0	6.64	7.9	58.8	54.37	6.515	4.73	0	0.75
Mean	2.009066	0.885042	0	0.591385	0.6295	2.028258	0.2555	3.150697	5.239	7.049329	2.619	5.265548	11.6665	10.19104	0.004	0.644732	0.001	1.003373	5.8505	7.993653	58.4895	54.40251	6.828	5.013551	0	1.150612
3rd Quantile	1.73464	0	0	0.96	0.91	2.68	0.1525	4.46	7.395	9.84	2.925	8.23	14.8775	13.51	0	0.63	0	1.37	7.465	9.28	63.4	61.35	7.18	6.41	0	1.96
Maximum	14.05094	22.57358	0	12.25	1.8	32.28	1.73	12.69	14.14	24.61	12.45	24.05	25.08	40.72	0.08	15.89	0.02	16.79	8.87	17.51	68.97	90.14	18.01	17.26	0	7.63

Supplementary Table 1. Summary statistics of the deconvolved cell types in blood samples.

	FCO (%)		Endothelial (%)		Stromal (%)		Astrocyte (%)		Microglial (%)		Oligodendrocyte (%)		GABA (%)		GABA (%)	
	DS	Normal	DS	Normal	DS	Normal	DS	Normal	DS	Normal	DS	Normal	DS	Normal	DS	Normal
Frontal cortex																
Minium	15.33376	15.36176	4.84733	3.558723	1.67267	1.679278	7.785059	5.338743	12.44571	8.136767	23.29629	21.47806	6.870092	1.672873	20.34485	7.797127
1st Quantile	18.73957	18.45195	6.707596	4.315457	2.914188	2.054543	9.773215	7.049398	14.83741	10.50494	25.46827	33.88208	8.688886	4.665274	20.92197	22.32381
Median	19.93851	22.73259	7.600056	4.786847	3.380172	2.533153	10.70272	7.508571	17.73644	11.33512	28.47084	35.17067	9.420401	5.311345	21.56517	26.35743
Mean	19.83307	21.40743	7.459854	5.512225	3.260146	2.832481	10.69098	8.662901	17.07222	12.21682	28.8618	39.3591	8.981225	5.667057	23.63877	24.56353
3rd Quantile	21.03201	23.41544	8.352315	5.609263	3.72613	3.410956	11.62048	9.401773	19.97125	13.04949	31.86437	46.48881	9.71274	6.256673	24.28197	28.66143
Maximum	24.12152	27.64763	9.791972	13.04631	4.607571	6.593691	13.57342	19.5689	20.37029	24.42304	35.20923	68.90632	10.21401	10.44045	31.07991	34.07955
Frontal cortex whole gray matter																
Minium	12.67364	18.64614	3.001274	2.41	0.017757	0	3.996832	5.783978	9.028724	8.096828	32.97289	36.67921	3.007764	1.904526	20.5607	21.83892
1st Quantile	18.49241	26.29747	3.534881	3.44154	0.24963	0.16416	4.893639	6.295501	10.24652	10.5265	38.01213	38.97768	4.049272	2.328068	22.58972	23.2319
Median	20.04519	27.73786	3.830826	3.999888	0.490562	0.373712	5.516039	6.424405	11.74645	10.93734	39.39564	42.0082	4.480441	2.77045	25.94131	24.74621
Mean	20.09494	27.75092	4.417921	4.034337	0.504222	0.520663	5.878026	7.207281	11.71702	11.05942	40.25495	41.8533	4.706071	2.796412	25.4875	24.94359
3rd Quantile	22.70673	31.53945	5.370419	4.284725	0.535755	0.602775	7.012655	7.90349	13.01824	11.64709	42.63576	43.30752	5.632175	3.136446	28.55681	26.80212
Maximum	25.6212	33.62901	6.64	6.710939	1.66	1.799061	7.811296	10.63194	14.44531	13.67796	48.24287	49.42496	6.538844	3.801078	30.55356	28.54688
Frontal cortex neuron																
Minium	9.402631	12.95972	0	0	0	0	1.53702	0.707038	0.864398	0.218011	9.309207	10.65795	8.306068	9.822709	33.56	57.20757
1st Quantile	14.04527	17.59681	0.285732	0	0.150547	0	2.406055	0.990771	1.331025	0.926948	11.29955	11.28747	12.60424	10.96599	51.50115	60.4035
Median	17.35928	18.0767	0.579091	0	0.219311	0	3.059834	1.445517	2.466532	1.43843	17.45558	12.77179	15.5	13.3687	52.47576	62.48387
Mean	16.83841	18.4122	1.220867	0.6867	0.586911	0.3301	3.957626	1.448277	3.464267	1.359967	16.29477	13.36357	15.0213	13.11957	53.2787	62.67225
3rd Quantile	19.29694	18.88273	0.789453	0.6166	0.424268	0.242	5.144912	1.518518	5.337831	1.738676	20.64919	14.6261	16.73885	14.53252	57.42004	64.16681
Maximum	25.54267	22.61974	6.87347	6.8735	3.06653	3.0665	9.339499	3.255658	8.720857	2.328068	22.93964	18.61008	21.94996	17.35126	66.59579	68.03729
Frontal cortex glia																
Minium	9.286262	13.48497	2.548926	1.2773	2.111074	1.3027	4.47779	2.991963	13.15084	8.318343	47.90443	63.20617	2.335177	1.699505	3.254823	2.410932
1st Quantile	10.92828	27.49433	3.350404	1.736601	2.149596	1.577647	4.640146	4.233594	14.42184	9.34435	56.55676	68.61899	2.856503	1.921871	3.547978	2.812603
Median	15.2359	27.65511	3.562655	2.040854	3.053267	1.740651	5.41411	4.305621	15.61229	10.52174	63.97749	72.75288	3.105538	2.098943	3.854462	3.310495
Mean	15.40399	26.68932	3.854597	2.323503	2.965403	2.042211	5.615802	4.353601	18.33331	12.22009	61.412	71.22917	3.124165	2.11738	4.069168	3.755477
3rd Quantile	16.58251	29.2275	4.068542	3.064695	3.181458	2.616405	5.910227	4.56456	21.77474	14.56356	68.40536	74.12802	3.392022	2.336469	4.043497	3.929398
Maximum	25.45812	32.2415	6.344337	3.343776	4.055663	2.864023	8.947083	5.581315	30.5131	18.88471	70.99488	77.15112	3.834444	2.506531	6.67095	7.082913
Cerebellum																
Minium	15.90475	4.083488	0	0	0	0	8.870641	8.54195	12.12028	10.21081	34.33868	35.13673	1.866359	0.793875	24.94364	19.09926
1st Quantile	16.32846	9.04286	0	0	0	0	9.185813	8.809686	12.4054	10.9488	34.56988	37.51627	2.246547	2.268882	25.74845	22.42159
Median	17.94119	12.18089	0	0	0	0	9.463178	8.919744	13.00309	11.12192	34.93301	38.12614	3.105801	2.860743	26.02353	22.55796
Mean	17.98341	11.6037	0	0	0	0	9.561161	9.794863	13.02351	11.34619	35.17783	38.05894	3.179407	3.085344	25.82809	22.4491
3rd Quantile	19.59614	14.48095	0	0	0	0	9.838525	10.81888	13.6212	11.49952	35.54096	38.7095	4.03866	4.467598	26.10317	23.64613
Maximum	20.14653	17.72977	0	0	0	0	10.44765	12.94645	13.96757	13.1344	36.50661	40.11773	4.639666	5.342037	26.32168	23.78967
Cerebellar cortex whole gray matter																
Minium	12.53096	6.934888	0	0	0	0	6.687191	7.729021	8.378232	8.806652	33.00353	33.78459	1.244391	1.691844	22.44146	20.86121
1st Quantile	15.73444	8.47285	0	0	0	0	7.75429	8.011571	11.0576	10.27943	36.69465	37.03629	3.355781	2.606727	25.32537	23.03469
Median	16.29812	13.45987	0	0	0	0	8.019955	8.14469	11.48791	11.17363	37.39895	37.7245	3.859162	3.845048	26.11265	23.30928
Mean	17.19752	12.9597	0	0	0	0	8.004823	8.159124	11.28273	10.79417	37.03244	37.2367	3.751829	3.642796	25.99942	23.30951
3rd Quantile	18.15916	14.85875	0	0	0	0	8.167229	8.336965	11.93977	11.54998	38.3516	38.30448	4.312636	4.336587	27.32155	23.91727
Maximum	25.14644	24.53263	0	0	0	0	9.235743	8.656354	12.87795	12.1064	39.69486	39.03216	5.297556	5.865314	27.70416	25.4685
Cerebrum																
Minium	32.29239	40.08368	10.64496	12.171	2.040217	3.178757	4.335586	4.31596	5.441826	6.424444	35.78787	37.34562	8.061185	8.706003	10.19649	7.99785
1st Quantile	36.11469	43.13236	12.32947	13.53525	2.932558	3.620072	4.456414	4.522779	6.006758	7.165953	37.69806	40.76899	8.467274	9.18932	11.14245	10.55021
Median	47.01493	53.62467	12.75252	15.17436	5.80114	4.597629	4.616608	4.634853	7.144075	8.254197	43.55928	42.74912	9.588621	9.248255	13.15609	12.16031
Mean	57.27216	64.0449	13.05279	14.74428	5.447214	5.140718	4.716865	4.786578	7.567997	8.185268	42.11139	42.31315	11.78304	10.91117	12.75071	11.6955
3rd Quantile	80.11344	86.38226	13.70678	15.7254	7.902587	6.794583	4.862982	4.708447	9.197961	8.847331	45.73818	44.78582	15.04005	11.95063	13.93278	12.96784
Maximum	97.0751	100	15.73246	17.04802	8.180134	7.64917	5.533349	5.915404	10.17418	10.33052	46.69405	45.46139	18.81351	16.2434	15.02691	14.604

Supplementary Table 2. Summary statistics of the deconvolved cell types in brain samples.

Buccal Swab	FCO (%)		Epithelial (%)		Fibroblast (%)		Immune cell (%)	
	DS	Normal	DS	Normal	DS	Normal	DS	Normal
Minium	0	0	33.104	71.10579	0	0	5.482173	3.639174
1st Quantile	4.396766	0	47.93361	83.4632	0	0	11.06411	5.849396
Median	7.133024	0	69.29672	90.98255	0	0	30.70328	9.017448
Mean	8.163798	1.543423	68.07432	87.86697	0.228146	0	31.69753	12.13303
3rd Quantile	11.92548	0.885742	88.93589	94.1506	0	0	52.06639	16.5368
Maximum	20.63272	11.20508	94.51783	96.36083	2.281464	0	64.61453	28.89421

Supplementary Table 3. Summary statistics of the deconvolved cell types in buccal swab samples.

	estimate	std.error	t-statistic	p.value	FDR
**FCO**	**-17.46**	**4.59**	**-3.8**	**1.90E-04**	**5.70E-04**
**Bas**	**-2.69**	**1.08**	**-2.49**	**1.40E-02**	**2.60E-02**
Bmem	-0.25	0.24	-1.05	2.90E-01	3.40E-01
**Bnv**	**-4.75**	**1.26**	**-3.77**	**2.20E-04**	**5.70E-04**
**CD4mem**	**1.24**	**0.27**	**4.63**	**7.00E-06**	**3.00E-05**
CD4nv	-1.9	1.58	-1.21	2.30E-01	3.00E-01
**CD8mem**	**7.61**	**0.94**	**8.07**	**9.00E-14**	**1.20E-12**
**CD8nv**	**-2.73**	**0.96**	**-2.85**	**4.90E-03**	**1.10E-02**
**Eos**	**-1.47**	**0.63**	**-2.33**	**2.10E-02**	**3.40E-02**
Mono	-0.45	0.6	-0.75	4.60E-01	5.00E-01
Neu	0.65	3.6	0.18	8.60E-01	8.60E-01
**NK**	**4.64**	**0.95**	**4.87**	**2.40E-06**	**1.60E-05**
**Treg**	**-1.41**	**0.64**	**-2.22**	**2.80E-02**	**4.00E-02**

Supplementary Table 4. Multiple variable linear regression model outputs for blood cell change in proportion (%) with DS adjusting for sex and age in the age group 0-5 (DS N=17; Normal N=171).

	estimate	std.error	t-statistic	p.value	FDR
FCO	1.4	1.41	0.99	3.20E-01	3.50E-01
**Bas**	**-1.14**	**0.45**	**-2.51**	**1.30E-02**	**2.40E-02**
Bmem	-0.11	0.28	-0.4	6.90E-01	6.90E-01
**Bnv**	**-5.25**	**0.88**	**-6**	**2.50E-08**	**1.60E-07**
**CD4mem**	**2.56**	**0.71**	**3.63**	**4.20E-04**	**1.10E-03**
**CD4nv**	**-7.92**	**1.05**	**-7.57**	**1.10E-11**	**1.40E-10**
**CD8mem**	**5.54**	**1.44**	**3.85**	**2.00E-04**	**6.50E-04**
**CD8nv**	**-3.96**	**0.92**	**-4.28**	**3.90E-05**	**1.70E-04**
Eos	-0.74	0.5	-1.49	1.40E-01	1.80E-01
Mono	-0.91	0.73	-1.25	2.10E-01	2.50E-01
Neu	5.8	2.93	1.98	5.00E-02	8.10E-02
NK	1.61	0.89	1.82	7.20E-02	1.00E-01
**Treg**	**-2.24**	**0.76**	**-2.97**	**3.70E-03**	**8.00E-03**

Supplementary Table 5. Multiple variable linear regression model outputs for blood cell change in proportion (%) with DS adjusting for sex and age in the age group 10-18 (DS N=9; Normal N=108).

	estimate	std.error	t-statistic	p.value	FDR
FCO	1.34	0.67	1.99	4.70E-02	6.00E-02
**Bas**	**-0.78**	**0.2**	**-3.82**	**1.40E-04**	**3.00E-04**
**Bmem**	**-0.93**	**0.37**	**-2.52**	**1.20E-02**	**2.20E-02**
**Bnv**	**-4.02**	**0.5**	**-8.03**	**2.30E-15**	**3.00E-14**
CD4mem	0.4	0.96	0.41	6.80E-01	6.80E-01
**CD4nv**	**-4.26**	**0.86**	**-4.94**	**9.10E-07**	**5.90E-06**
CD8mem	1.99	1.36	1.47	1.40E-01	1.50E-01
**CD8nv**	**-1.4**	**0.29**	**-4.78**	**2.00E-06**	**8.70E-06**
**Eos**	**-0.88**	**0.4**	**-2.17**	**3.00E-02**	**4.30E-02**
**Mono**	**-2.05**	**0.49**	**-4.21**	**2.70E-05**	**7.00E-05**
Neu	4.51	2.31	1.95	5.10E-02	6.00E-02
**NK**	**1.36**	**0.56**	**2.41**	**1.60E-02**	**2.60E-02**
**Treg**	**-1.23**	**0.29**	**-4.25**	**2.30E-05**	**7.00E-05**

Supplementary Table 6. Multiple variable linear regression model outputs for blood cell change in proportion (%) with DS adjusting for sex and age in the age group >18 (DS N=20; Normal N=1180).

	estimate	std.error	t-statistic	p.value	FDR
FCO	-0.9	1.87	-0.48	6.40E-01	7.30E-01
Endothelial	2.97	1.25	2.38	3.00E-02	6.00E-02
Stromal	1.03	0.66	1.57	1.40E-01	1.90E-01
Astrocyte	3.48	1.74	2	6.20E-02	9.90E-02
**Microglial**	**6.32**	**2.15**	**2.94**	**9.10E-03**	**2.90E-02**
**Oligodendrocyte**	**-16.77**	**5.91**	**-2.84**	**1.10E-02**	**2.90E-02**
**GABA**	**4.44**	**1.01**	**4.42**	**3.80E-04**	**3.00E-03**
GLU	0.26	4.33	0.06	9.50E-01	9.50E-01

Supplementary Table 7. Multiple variable linear regression model outputs for brain cell change in proportion (%) with DS adjusting for sex and age in the frontal cortex (DS N=4; Normal N=17).

	estimate	std.error	t-statistic	p.value	FDR
**FCO**	**-4.72**	**1.58**	**-2.98**	**8.00E-03**	**3.20E-02**
Endothelial	0.72	0.6	1.21	2.40E-01	4.80E-01
Stromal	0.15	0.23	0.65	5.20E-01	6.90E-01
Astrocyte	-0.92	0.7	-1.31	2.10E-01	4.80E-01
Microglial	0.64	0.94	0.68	5.00E-01	6.90E-01
Oligodendrocyte	-0.91	2.09	-0.44	6.70E-01	7.00E-01
**GABA**	**1.64**	**0.43**	**3.78**	**1.40E-03**	**1.10E-02**
GLU	-0.59	1.53	-0.39	7.00E-01	7.00E-01

Supplementary Table 8. Multiple variable linear regression model outputs for brain cell change in proportion (%) with DS adjusting for sex and age in the frontal cortex whole gray matter (DS N=14; Normal N=8).

	estimate	std.error	t-statistic	p.value	FDR
FCO	-1.52	1.63	-0.94	3.60E-01	3.60E-01
Endothelial	1.83	0.85	2.15	5.30E-02	8.50E-02
Stromal	0.86	0.39	2.22	4.60E-02	8.50E-02
**Astrocyte**	**2.56**	**0.79**	**3.24**	**5.20E-03**	**3.60E-02**
Microglial	2.14	0.86	2.49	2.40E-02	6.40E-02
Oligodendrocyte	3.08	1.84	1.67	1.10E-01	1.50E-01
GABA	1.7	1.62	1.05	3.10E-01	3.50E-01
**GLU**	**-9.41**	**3.16**	**-2.97**	**9.00E-03**	**3.60E-02**

Supplementary Table 9. Multiple variable linear regression model outputs for brain cell change in proportion (%) with DS adjusting for sex and age in the frontal cortex neuron (DS N=9; Normal N=7).

	estimate	std.error	t-statistic	p.value	FDR
**FCO**	**-8.56**	**2.78**	**-3.08**	**9.50E-03**	**3.80E-02**
Endothelial	1.34	0.62	2.17	5.10E-02	9.50E-02
Stromal	0.68	0.37	1.84	9.00E-02	1.00E-01
Astrocyte	1.25	0.6	2.08	6.00E-02	9.50E-02
Microglial	6.01	3.03	1.98	7.10E-02	9.50E-02
Oligodendrocyte	-9.87	4.13	-2.39	3.40E-02	9.10E-02
**GABA**	**1.03**	**0.21**	**4.89**	**3.70E-04**	**3.00E-03**
GLU	0.87	0.68	1.28	2.30E-01	2.30E-01

Supplementary Table 10. Multiple variable linear regression model outputs for brain cell change in proportion (%) with DS adjusting for sex and age in the frontal cortex glia (DS N=9; Normal N=11).

	estimate	std.error	t-statistic	p.value	FDR
FCO	5.87	3.68	1.59	1.50E-01	2.20E-01
Endothelial	0	0	NA	NaN	NaN
Stromal	0	0	NA	NaN	NaN
Astrocyte	-0.93	1.26	-0.74	4.80E-01	5.80E-01
Microglial	1.59	0.81	1.96	8.20E-02	1.60E-01
**Oligodendrocyte**	**-3.9**	**1.2**	**-3.25**	**1.00E-02**	**3.00E-02**
GABA	0.4	1.39	0.29	7.80E-01	7.80E-01
**GLU**	**5.06**	**1.02**	**4.96**	**7.80E-04**	**4.70E-03**

Supplementary Table 11. Multiple variable linear regression model outputs for brain cell change in proportion (%) with DS adjusting for sex and age in the cerebellum (DS N=4; Normal N=9).

	estimate	std.error	t-statistic	p.value	FDR
**FCO**	**6.85**	**1.92**	**3.56**	**1.50E-03**	**9.00E-03**
Endothelial	0	0	NA	NaN	NaN
Stromal	0	0	NA	NaN	NaN
Astrocyte	-0.3	0.25	-1.23	2.30E-01	4.60E-01
Microglial	0.48	0.61	0.79	4.40E-01	5.30E-01
Oligodendrocyte	-0.85	0.96	-0.88	3.90E-01	5.30E-01
GABA	0	0.63	0	1.00E+00	1.00E+00
**GLU**	**2.21**	**0.71**	**3.1**	**4.70E-03**	**1.40E-02**

Supplementary Table 12. Multiple variable linear regression model outputs for brain cell change in proportion (%) with DS adjusting for sex and age in the cerebellar cortex whole gray matter (DS N=16; Normal N=13).

	estimate	std.error	t-statistic	p.value	FDR
FCO	-2.24	15.41	-0.15	8.90E-01	9.90E-01
Endothelial	-1.11	0.89	-1.24	2.40E-01	9.90E-01
Stromal	0.81	1.45	0.56	5.90E-01	9.90E-01
Astrocyte	0	0.26	0.02	9.90E-01	9.90E-01
Microglial	-0.38	1.04	-0.36	7.20E-01	9.90E-01
Oligodendrocyte	-0.47	2.47	-0.19	8.50E-01	9.90E-01
GABA	0.72	2.4	0.3	7.70E-01	9.90E-01
GLU	0.75	1.25	0.6	5.60E-01	9.90E-01

Supplementary Table 13. Multiple variable linear regression model outputs for brain cell change in proportion (%) with DS adjusting for sex and age in the fetal cerebrum (DS N =8; Normal N =6).

	estimate	std.error	t-statistic	p.value	FDR
**FCO**	**6.53**	**2.24**	**2.92**	**1.00E-02**	**1.30E-02**
**Epithelial cell**	**-20.2**	**6.9**	**-2.93**	**9.90E-03**	**1.30E-02**
Fibroblast	0.24	0.2	1.23	2.40E-01	2.40E-01
**Immune cell**	**19.96**	**6.85**	**2.92**	**1.00E-02**	**1.30E-02**

Supplementary Table 14. Multiple variable linear regression model outputs for buccal swab cell change in proportion (%) with DS adjusting for sex and age (DS N=10; Normal N=10).

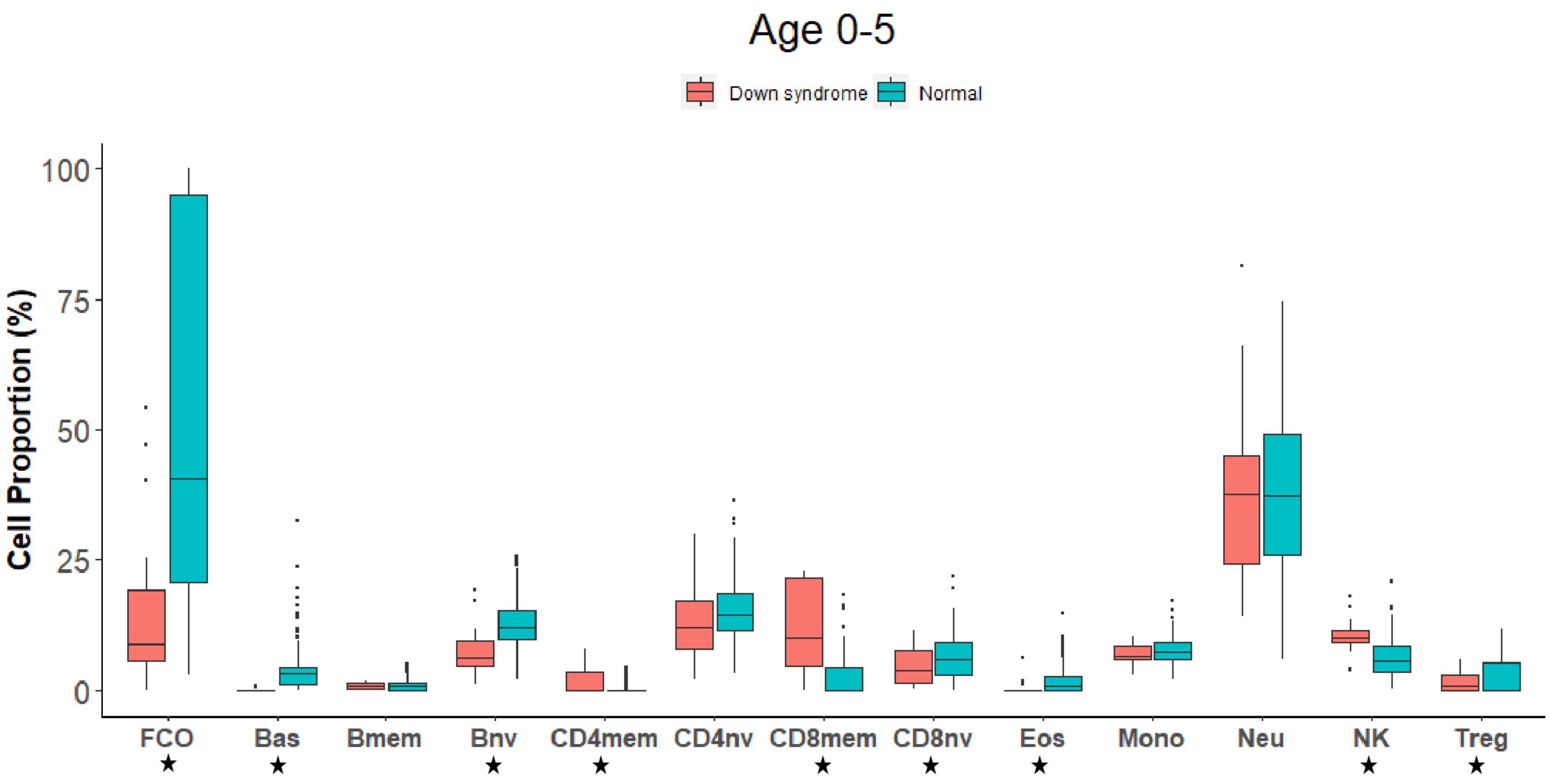

Supplementary Figure 1. Boxplots on blood cell differences between DS and normal groups in the age group 0-5 (DS N=17; Normal N=171). The lines in boxplots represent the upper quartile, median, and lower quartile from top to bottom. The star indicates statistical significance (FDR < 0.05 with sex and age adjusted).

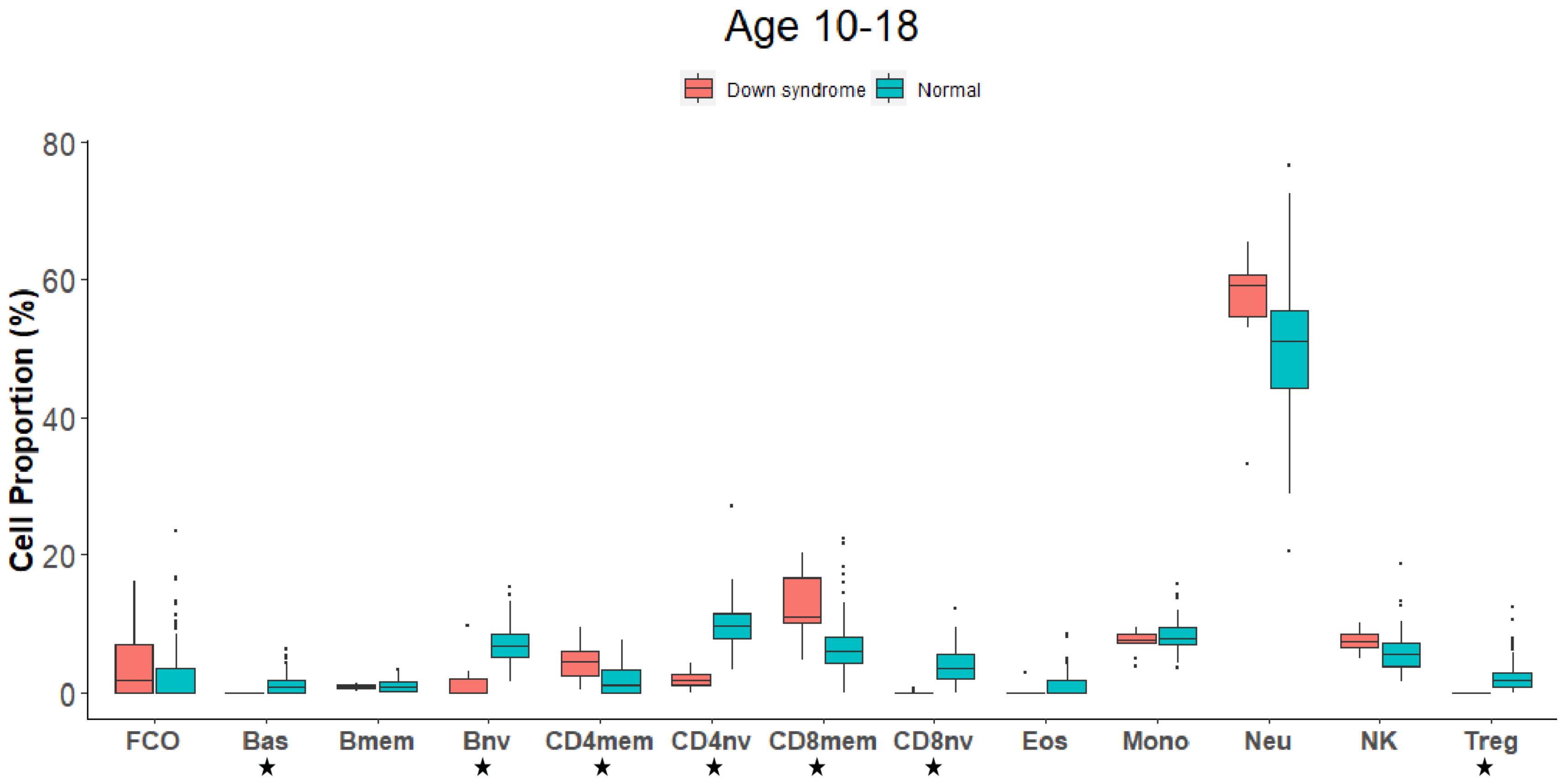

Supplementary Figure 2. Boxplots on blood cell differences between DS and normal groups in the age group 10-18 (DS N=9; Normal N=108). The lines in boxplots represent the upper quartile, median, and lower quartile from top to bottom. The star indicates statistical significance (FDR < 0.05 with sex and age adjusted).

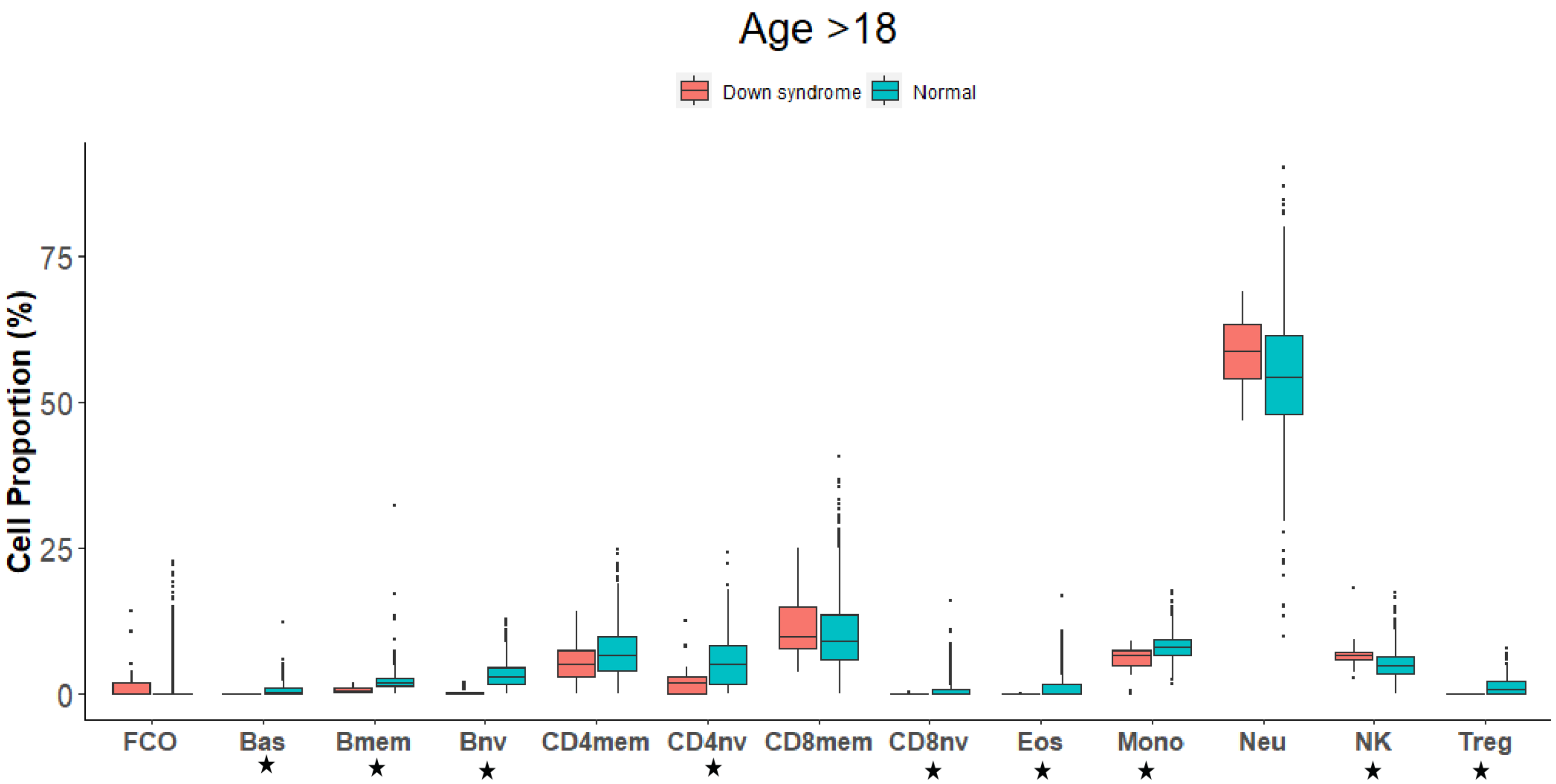

Supplementary Figure 3. Boxplots on blood cell differences between DS and normal groups in the age group >18 (DS N=20; Normal N=1180). The lines in boxplots represent the upper quartile, median, and lower quartile from top to bottom. The star indicates statistical significance (FDR < 0.05 with sex and age adjusted).

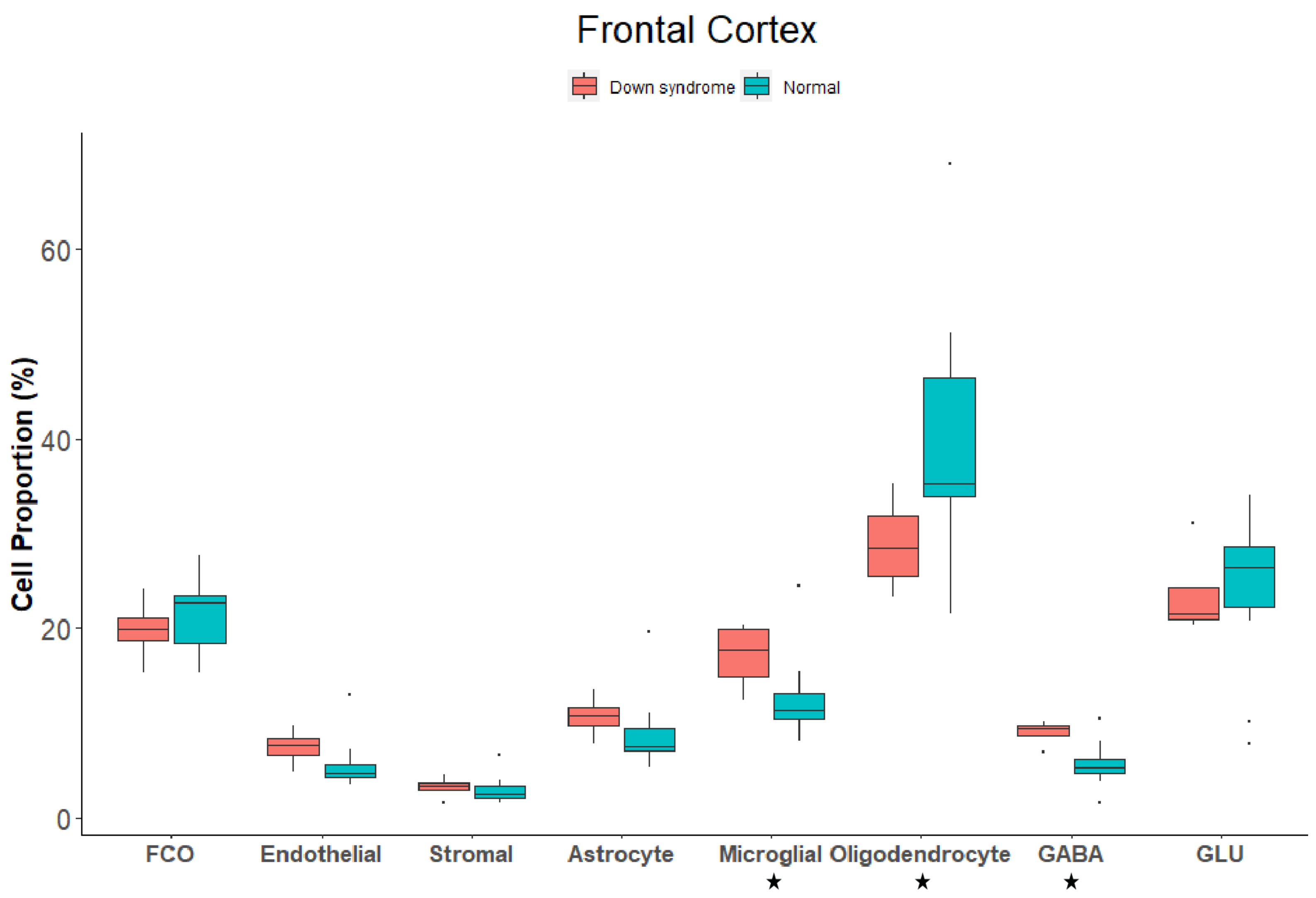

Supplementary Figure 4. Boxplots on brain cell differences between DS and normal groups in the frontal cortex (DS N=4; Normal N=17). The lines in boxplots represent the upper quartile, median, and lower quartile from top to bottom. The star indicates statistical significance (FDR < 0.05 with sex and age adjusted).

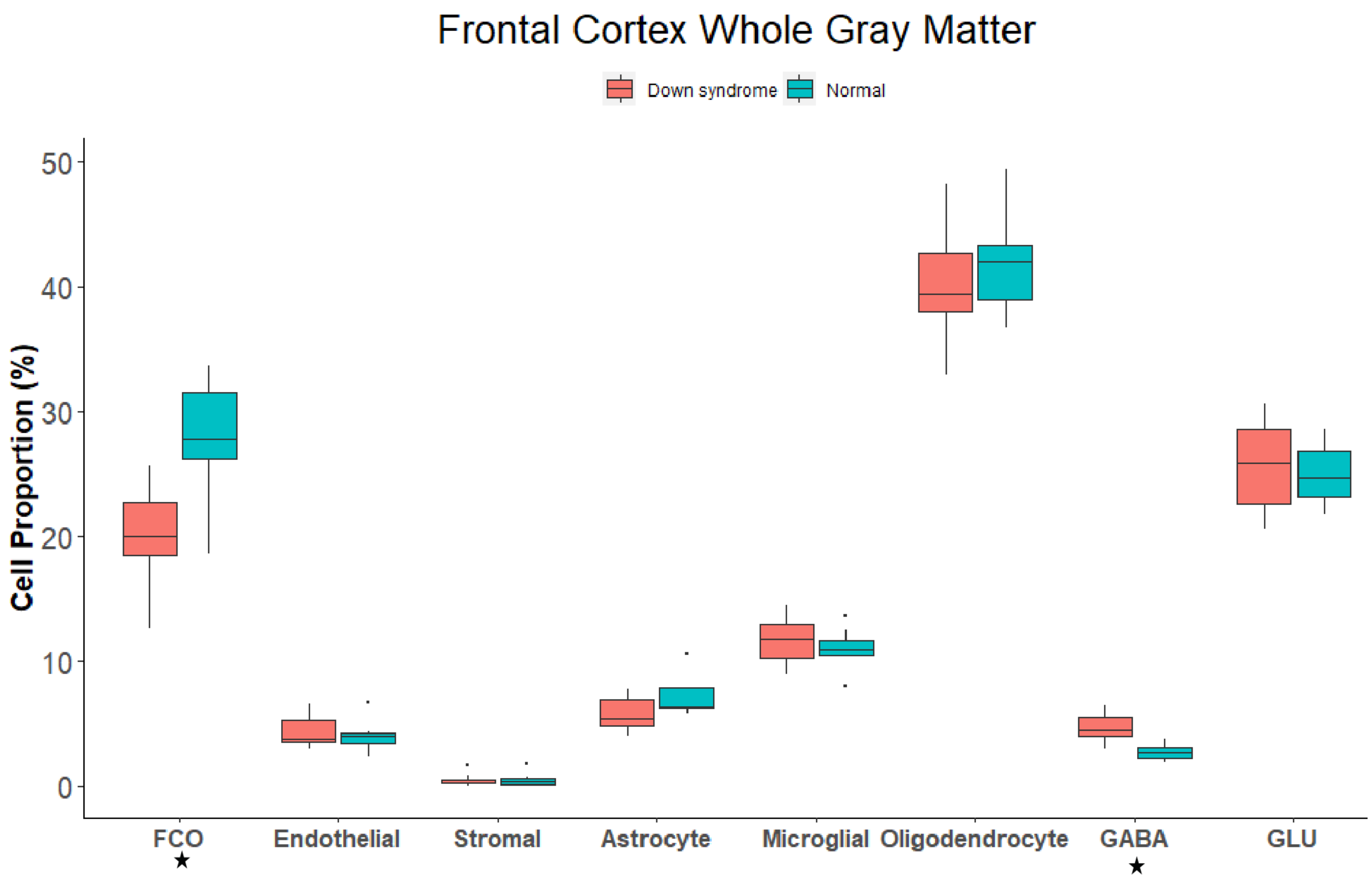

Supplementary Figure 5. Boxplots on brain cell differences between DS and normal groups in the frontal cortex whole gray matter (DS N=14; Normal N=8). The lines in boxplots represent the upper quartile, median, and lower quartile from top to bottom. The star indicates statistical significance (FDR < 0.05 with sex and age adjusted).

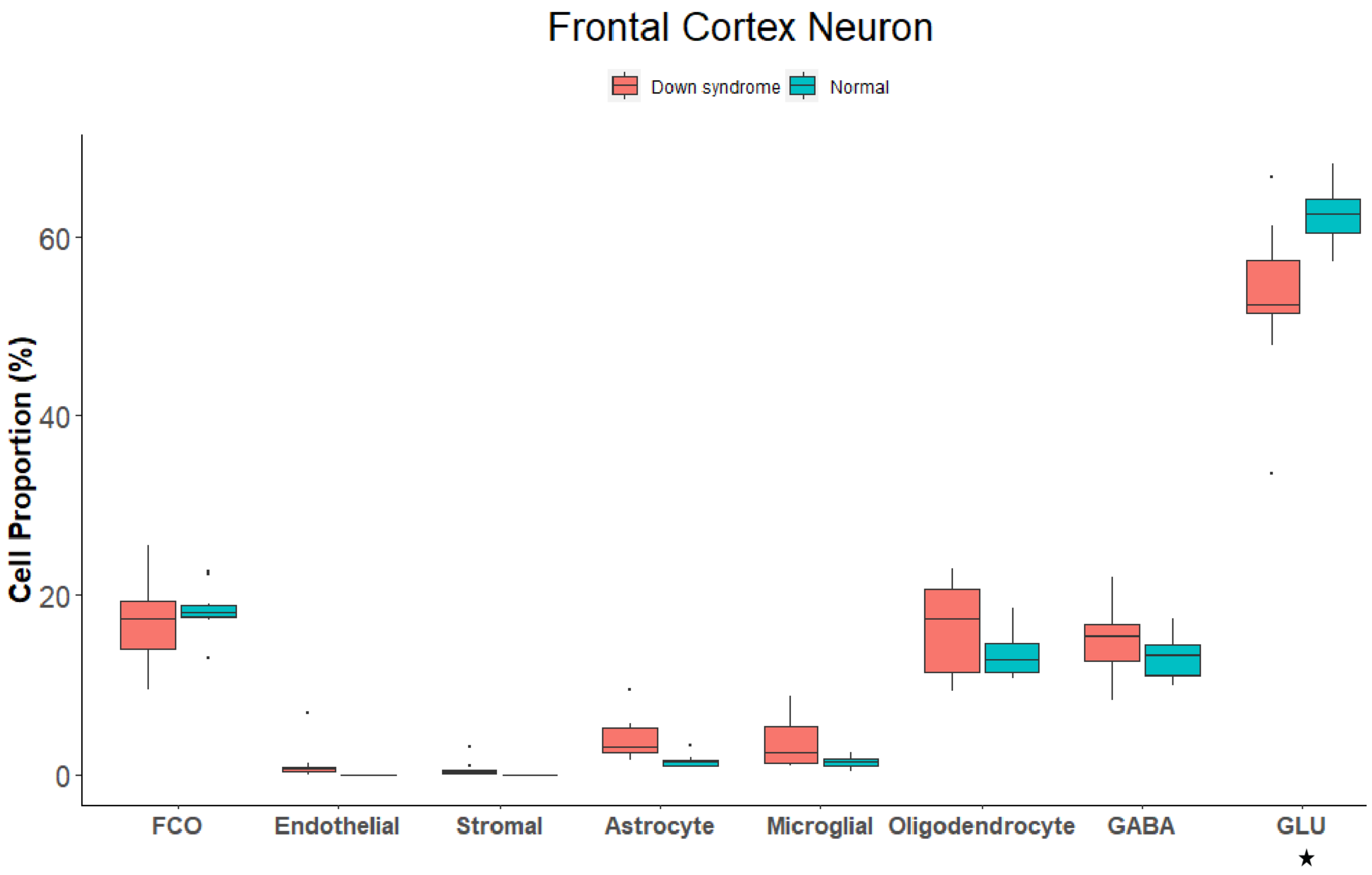

Supplementary Figure 6. Boxplots on brain cell differences between DS and normal groups in the frontal cortex neuron (DS N=9; Normal N=7). The lines in boxplots represent the upper quartile, median, and lower quartile from top to bottom. The star indicates statistical significance (FDR < 0.05 with sex and age adjusted).

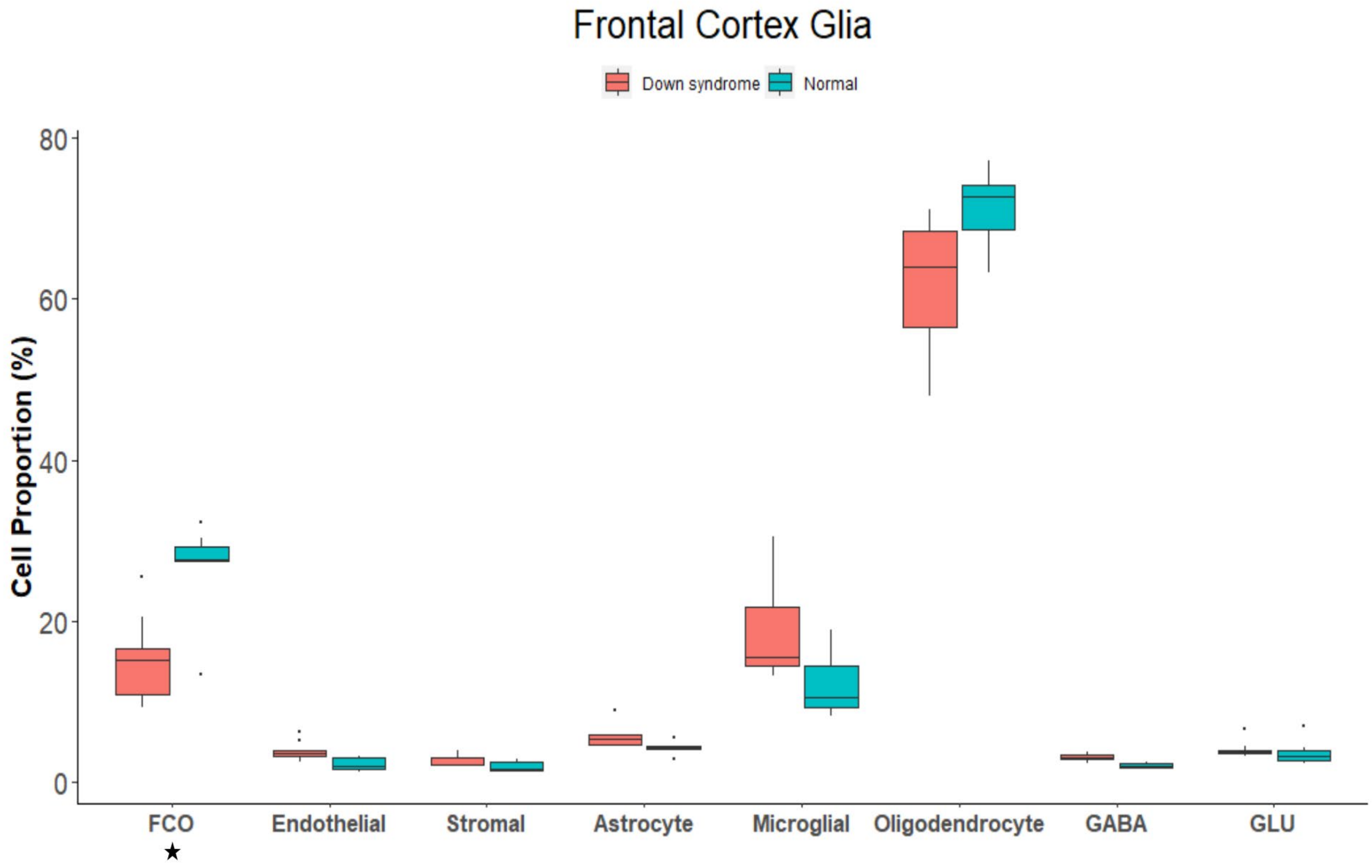

Supplementary Figure 7. Boxplots on brain cell differences between DS and normal groups in the frontal cortex glia (DS N=9; Normal N=11). The lines in boxplots represent the upper quartile, median, and lower quartile from top to bottom. The star indicates statistical significance (FDR < 0.05 with sex and age adjusted).

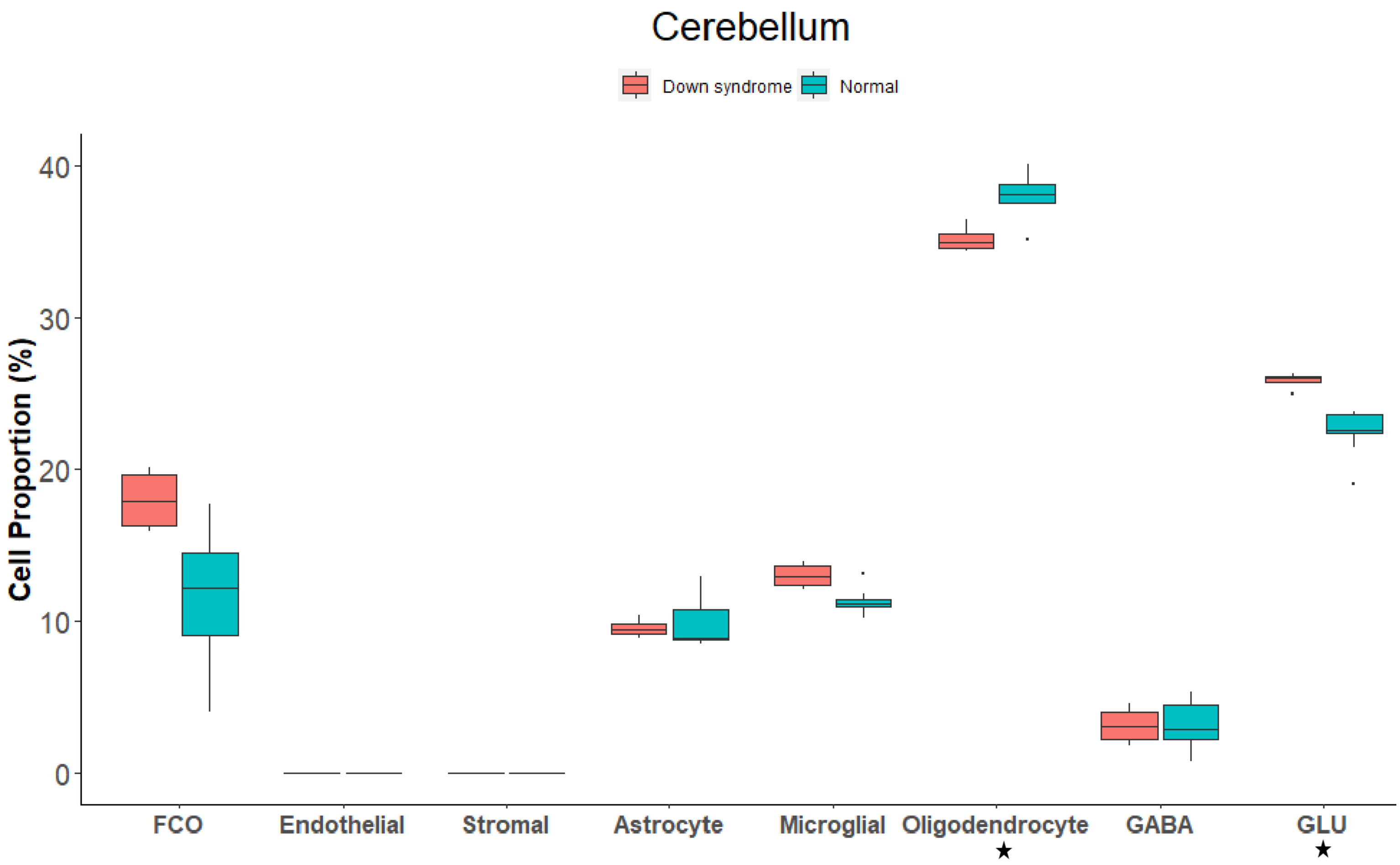

Supplementary Figure 8. Boxplots on brain cell differences between DS and normal groups in the cerebellum (DS N=4; Normal N=9). The lines in boxplots represent the upper quartile, median, and lower quartile from top to bottom. The star indicates statistical significance (FDR < 0.05 with sex and age adjusted).

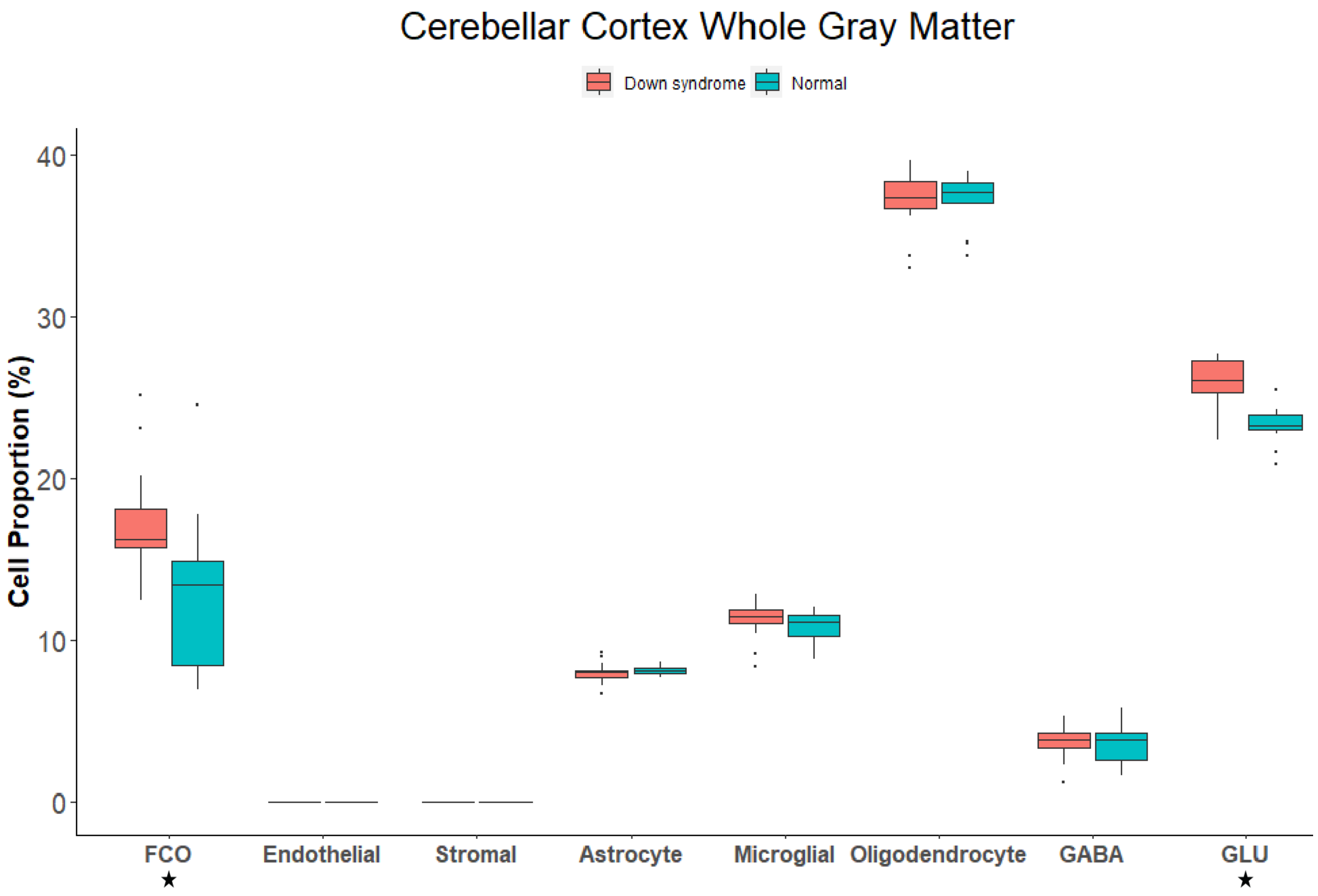

Supplementary Figure 9. Boxplots on brain cell differences between DS and normal groups in the cerebellar cortex whole gray matter (DS N=16; Normal N=13). The lines in boxplots represent the upper quartile, median, and lower quartile from top to bottom. The star indicates statistical significance (FDR < 0.05 with sex and age adjusted).

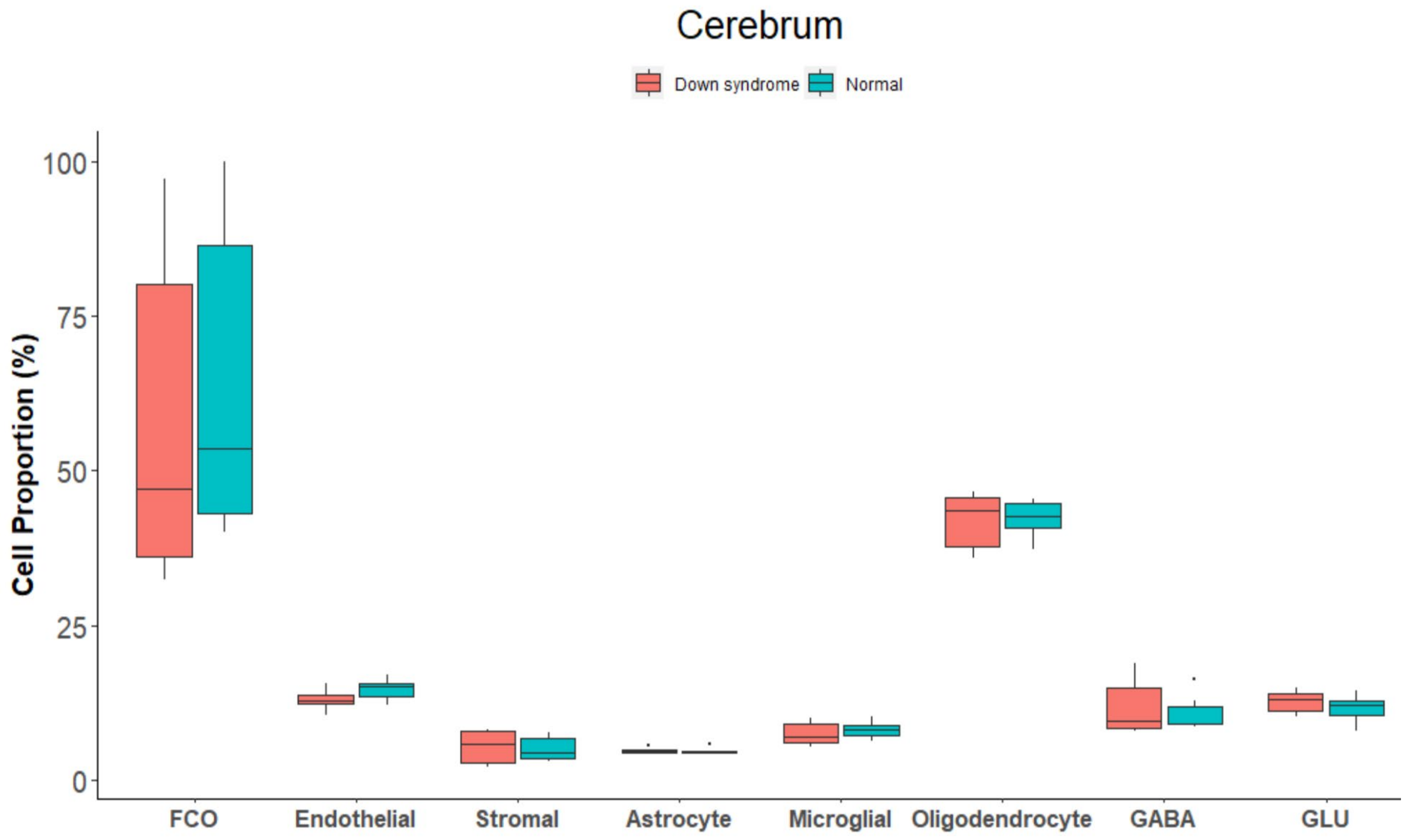

Supplementary Figure 10. Boxplots on brain cell differences between DS and normal groups in the fetal cerebrum (DS N =8; Normal N =6). The lines in boxplots represent the upper quartile, median, and lower quartile from top to bottom. The star indicates statistical significance (FDR < 0.05 with sex and age adjusted).

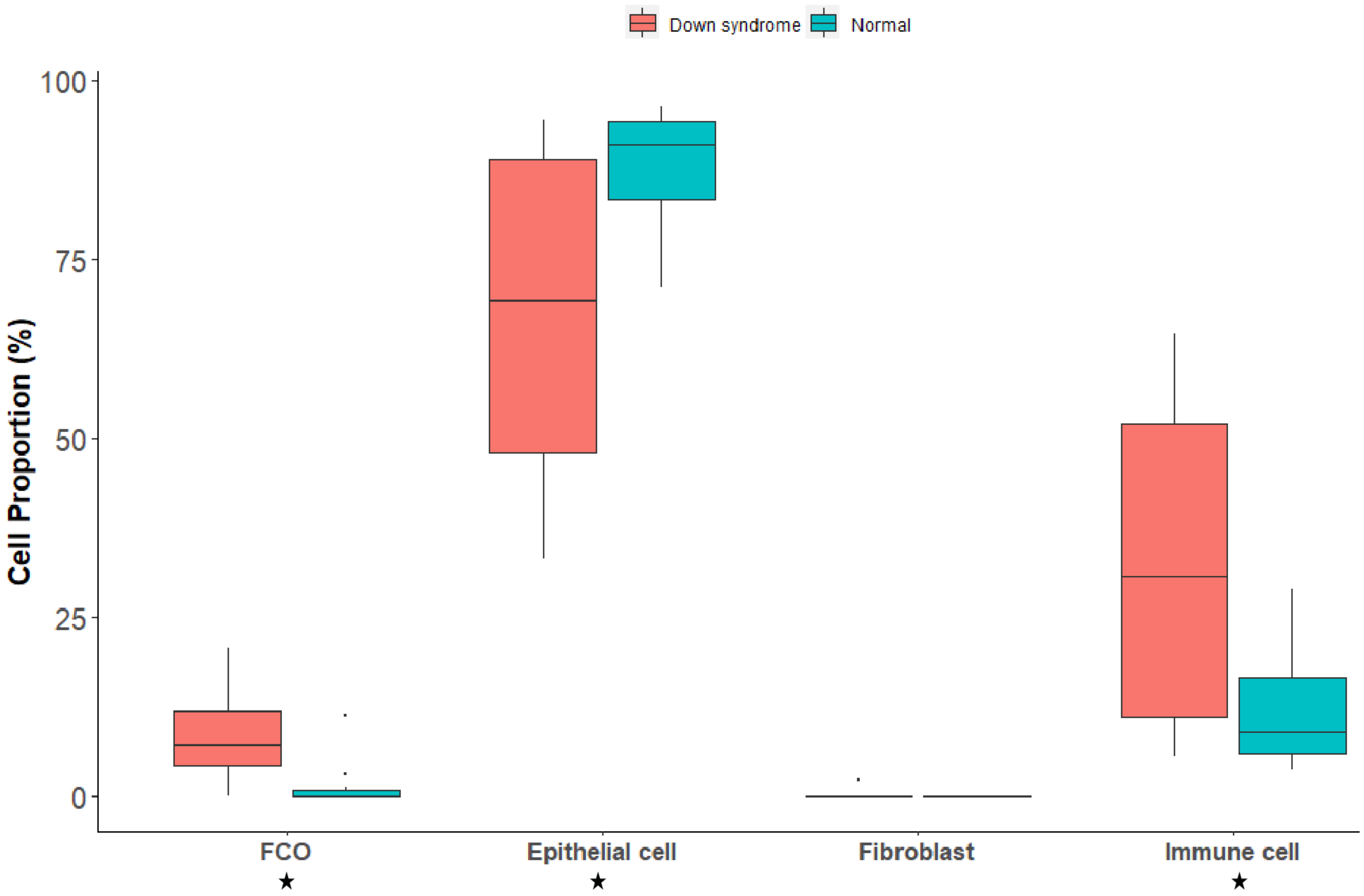

Supplementary Figure 11. Boxplots on brain cell differences between DS and normal groups in buccal swabs (DS N=10; Normal N=10). The lines in boxplots represent the upper quartile, median, and lower quartile from top to bottom. The star indicates statistical significance (FDR < 0.05 with sex and age adjusted).
